# Cell-Free DNA Hydroxymethylation in Cancer: Current and Emerging Detection Methods and Clinical Applications

**DOI:** 10.3390/genes15091160

**Published:** 2024-09-03

**Authors:** Janice J. N. Li, Geoffrey Liu, Benjamin H. Lok

**Affiliations:** 1 Department of Medical Biophysics, Temerty Faculty of Medicine, University of Toronto, Princess Margaret Cancer Research Tower, 101 College Street, Room 9-309, Toronto, ON M5G 1L7, Canada; 2 Institute of Medical Science, Temerty Faculty of Medicine, University of Toronto, 1 King’s College Circle, Medical Sciences Building, Room 2374, Toronto, ON M5S 1A8, Canada; 3 Department of Medical Oncology and Hematology, Princess Margaret Cancer Centre, 610 University Ave, Toronto, ON M5G 2C4, Canada; 4 Radiation Medicine Program, Princess Margaret Cancer Centre, 610 University Ave, Toronto, ON M5G 2C4, Canada

**Keywords:** 5-hydroxymethylcytosine, DNA hydroxymethylation, cell-free DNA, liquid biopsy, cancer biomarker, epigenetics

## Abstract

In the era of precision oncology, identifying abnormal genetic and epigenetic alterations has transformed the way cancer is diagnosed, managed, and treated. 5-hydroxymethylcytosine (5hmC) is an emerging epigenetic modification formed through the oxidation of 5-methylcytosine (5mC) by ten-eleven translocase (TET) enzymes. DNA hydroxymethylation exhibits tissue- and cancer-specific patterns and is essential in DNA demethylation and gene regulation. Recent advancements in 5hmC detection methods and the discovery of 5hmC in cell-free DNA (cfDNA) have highlighted the potential for cell-free 5hmC as a cancer biomarker. This review explores the current and emerging techniques and applications of DNA hydroxymethylation in cancer, particularly in the context of cfDNA.

## 1. Introduction

DNA hydroxymethylation (5-hydroxymethylcytosine, 5hmC) is an epigenetic modification that plays a fundamental role in biological processes such as normal development and gene regulation [1]. Dysregulation of 5hmC contributes to the development of various diseases, like cancer, highlighting the importance of understanding its biology [2,3]. Liquid biopsy provides a minimally invasive avenue for accessing genomic and epigenomic data, revolutionizing the way cancer is screened and treated [4]. By studying cell-free DNA (cfDNA), particularly circulating tumour DNA (ctDNA), from bodily fluids like blood plasma, it enables opportunities for capturing the heterogeneity of the tumour, monitoring genetic and epigenetic dynamics, and detecting early signs of cancer or recurrence [5]. As interest in cell-free 5hmC as a cancer biomarker is rising, it underscores the value of having sensitive and specific assays that can profile the hydroxymethylome for broad clinical insight [1,6,7,8].

In this review, we provide an overview of the biological roles and mechanisms of DNA hydroxymethylation, along with updated perspectives on recent advances in its profiling techniques and applications as a cancer biomarker. Additionally, we explore the use of cell-free DNA (cfDNA) and integrated, multi-omics analyses in 5hmC studies and discuss opportunities and challenges for their clinical utility.

## 2. Biological Functions and Mechanisms of DNA Hydroxymethylation

### 2.1. DNA Hydroxymethylation in the Demethylation Pathway

**Figure 1:**
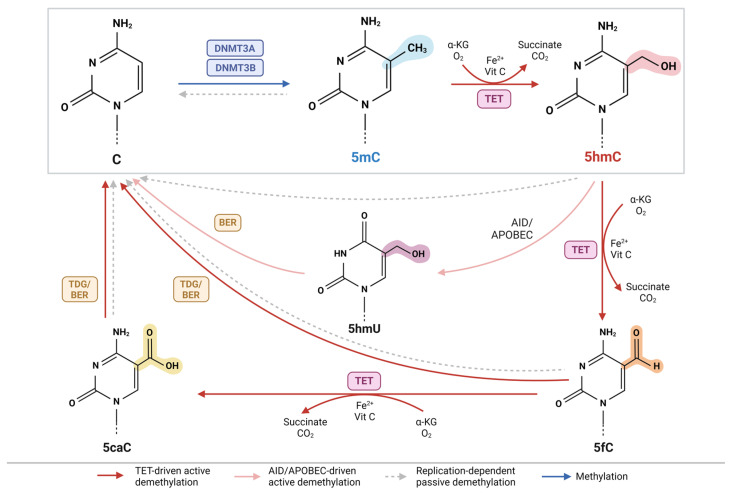
Active DNA Demethylation Pathway. 5-methylcytosine (5mC) can be actively demethylated by ten-eleven translocation (TET) enzymes to form 5-hydroxymethylcytosine (5hmC). TET requires alpha-ketoglutarate (α-KG) and oxygen (O_2_), in the presence of vitamin C (Vit C) and iron (Fe^2+^), to form succinate, carbon dioxide (CO_2_), and 5hmC. Further oxidation of 5hmC by TET yields 5-formylcytosine (5fC) and 5-carboxylcytosine (5caC). Both 5fC and 5caC can be excised and replaced by thymine DNA glycosylase (TDG) and base excision repair (BER) machinery, respectively, to return them to an unmethylated cytosine (C) form. Alternatively, 5hmC can be deaminated by Activation-Induced cytidine Deaminase/Apolipoprotein B mRNA Editing enzyme, Catalytic polypeptide-like (AID/APOBEC) enzymes into 5-hydroxymethyluracil (5hmU), which is subsequently returned to unmethylated C via BER. This figure was created with Biorender.com.

DNA methylation (5-methylcytosine, 5mC) is the most well-studied epigenetic marker in mammalian DNA and is important for gene regulation [9]. 5mC can be inherited or enzymatically produced through the covalent transfer of a methyl group from S-adenosylmethionine (SAM) onto the 5-position of cytosine by DNA methyltransferases (DNMT) [10]. DNA 5hmC is generated through the oxidation of 5mC by ten-eleven translocation (TET) dioxygenases, such as *TET1*, *TET2*, and *TET3*, during the active demethylation pathway (Figure 1) [11]. This reaction requires alpha-ketoglutarate (α-KG) and oxygen, in the presence of iron and ascorbic acid (vitamin C), to produce 5hmC, succinate, and carbon dioxide [12,13]. Further oxidation of 5hmC by TET enzymes yields the formation of 5-formylcytosine (5fC) and 5-carboxylcytosine (5caC), though these intermediates exist in much lower frequencies than 5hmC [12,14,15]. Unlike 5hmC, 5fC and 5caC are unstable and will be excised and repaired by thymine DNA glycosylase (TDG) and base excision repair (BER) machinery, respectively, restoring them to their unmodified cytosine form [16]. 5hmC can also be actively demethylated through activation-induced cytidine deaminase (AID)/apolipoprotein B mRNA editing enzyme, catalytic polypeptide-like (APOBEC) deamination into 5-hydroxymethyluracil (5mU), followed by BER [17]. Passive demethylation of cytosine modifications can also occur through replication dilution [18].

Interestingly, TET enzymes and TDG-BER coupled machinery exhibit a low affinity for 5hmC, compared to 5mC, 5fC, and 5caC, indicating that 5hmC is stable and not always readily demethylated [13,15,19,20]. Additionally, 5hmC can persist through multiple rounds of cell division, suggesting that it may have important biological functions beyond being an intermediate in the demethylation pathway [21].

### 2.2. Biological Functions and Distribution of DNA Hydroxymethylation

**Figure 2:**
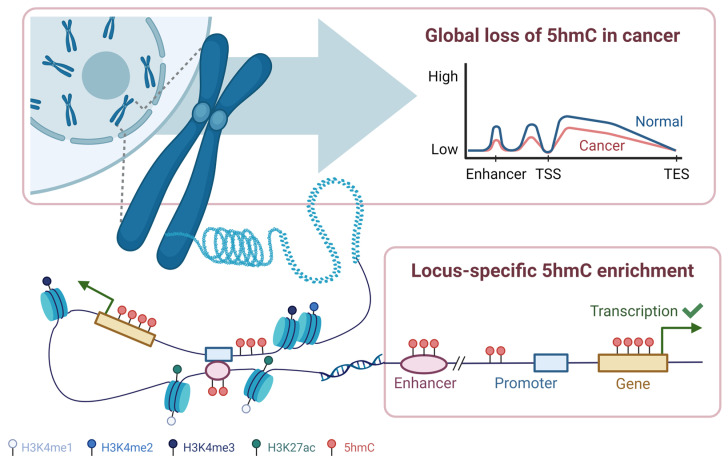
Global and Locus-Specific DNA Hydroxymethylation Patterns. DNA hydroxymethylation (5hmC) patterns can be measured globally or locally. In cancer, there is a reduction of 5hmC across the genome. On a locus-specific level, 5hmC tends to be enriched in transcriptionally active regions, such as at enhancers, the edge of promoters, and in the gene body. 5hmC can also be found near histones marking active gene expression or enhancer activity. In general, 5hmC levels are associated with active gene expression. TSS, transcription start site; TES, transcription end site; H3K4me1, histone H3 lysine 4 monomethylation; H3K4me2, histone H3 lysine 4 dimethylation; H3K4me3, histone H3 lysine 4 trimethylation; H3K4ac27, histone H3 lysine 4 acetylation. This figure was created with Biorender.com.

5hmC has implications in many biological processes, including embryonic development [22,23,24,25,26], stem cell pluripotency [11,27,28,29,30,31], T lymphocyte (T-cell) exhaustion and differentiation [32,33,34,35], and the pathogenesis of different diseases [2,13,36]. Importantly, 5hmC plays a pivotal role in active gene regulation. 5hmC is enriched in transcriptionally active regions, such as in gene bodies [32,37,38,39,40], at enhancers [37,38,41], and at the edge of promoters (Figure 2) [39,42,43,44]. 5hmC-rich regions are often located near active histone modifications (e.g., histone H3 lysine 4 dimethylation [H3K4me2] and histone H3 lysine 4 trimethylation [H3K4me3]) [23,39] and histone-marking enhancers (e.g., histone H3 lysine 4 monomethylation [H3K4me1] and histone H3 lysine 27 acetylation [H3K27ac]) [23,45], indicating its likely involvement in regulating active gene expression (Figure 2). Indeed, many studies have demonstrated a positive correlation between 5hmC levels and gene expression [23,37,38,40,46]. Though, depending on the hydroxymethylated region and cell-type involved, the opposite effect may be observed [47]. It is postulated that 5hmC regulates gene expression through several mechanisms, such as altering chromatin accessibility and inhibiting the binding affinity of repressive methyl-binding domain proteins, like methyl-CpG-binding protein 2 (MeCP2) [48,49,50]. However, this relationship is complex, as discussed elsewhere [45].

Despite low global 5hmC levels, the distribution of 5hmC varies greatly amongst different cell types, tissues, and disease states [37,50,51,52]. In general, 5hmC is most prevalent in brain tissue (~0.67% of all cytosines) and embryonic stem cells [53]. Interestingly, 5hmC abundance seems to be inversely correlated with cell proliferation, in which post-mitotic cells typically have higher levels of 5hmC [21]. This explains the low levels of 5hmC detected in cell lines [53,54] and tumour tissue [53,55,56,57] as these cells tend to be highly proliferative.

### 2.3. Cell-Free DNA and the Hydroxymethylome

Interest in investigating the cell-free hydroxymethylome was spurred in 2017 when Song and colleagues developed a highly sensitive chemical labeling assay capable of detecting low levels of 5hmC from small cfDNA inputs [58]. Since then, it has been explored in a wide variety of clinical applications, including early cancer detection [59,60,61,62] and predicting cancer outcomes [63,64,65,66]. Though 5hmC levels are typically lower in cfDNA compared to tissue, previous studies have revealed that cell-free 5hmC patterns and dynamics reflect changes that occur in tissue [59,67]. Moreover, since 5hmC is relatively stable, 5hmC marks on genomic DNA can be recapitulated in cfDNA fragments, suggesting its potential as a minimally-invasive biomarker for cancer [59].

### 2.4. DNA Hydroxymethylation Patterns in Cancer

DNA hydroxymethylation patterns can be assessed globally (across the genome) or locally (across specific gene features, Figure 2). Global loss of 5hmC has been detected across various types of cancers, including bladder, brain, pancreatic, and breast cancers [58,59,62,68,69,70,71]. In some cases, a greater reduction in 5hmC levels was associated with more aggressive tumours and poorer prognosis [58,69,72,73,74,75]. In cutaneous T-cell lymphoma (CTCL) cell lines, Qiu et al. (2018) observed gradual loss of 5hmC with increasing tumour aggressiveness, in which large cell-transformed CTCL cell lines exhibited the greatest percent 5hmC reduction, followed by non-transformed CTCL, and pre-CTCL plaques and patches [75]. Similarly, Song et al. (2017) found a stage-dependent depletion of 5hmC in cfDNA in non-small cell lung cancer (NSCLC), where metastatic NSCLC displayed the lowest levels of normalized hydroxymethylated regions, compared to non-metastatic NSCLC and healthy individuals [58]. However, it should be noted that these findings may vary depending on the tissue type and populations being studied [56,60,76]. For instance, unlike the study by Song et al. (2017), other NSCLC studies in Chinese cohorts reported global gains in 5hmC levels in cancer compared to healthy controls [60,76]. Nevertheless, global 5hmC patterns display distinct profiles between cancer and non-cancer controls.

Locus-specific 5hmC gains at transcriptionally active regions have also been described in cancer [59,60,77,78,79,80]. In a cfDNA study in colorectal cancer, Li et al. (2017) reported enrichment of 5hmC at gene bodies, open chromatin regions (DNase I hypersensitivity sites), and regions bearing active histone modifications (H3K27ac, H3K4me1, H3K9me1) [59]. In another study, Sjöstrom and colleagues (2022) saw that local 5hmC enrichment at enhancers or upstream of transcription start sites of prostate cancer drivers, most notably androgen receptor (*AR*) and forkhead box protein A1 (*FOXA1*), correlated with their gene expression [79]. This demonstrated that dysregulation of 5hmC could lead to disease states, even without genetic mutations at the driver [79].

Together, these studies indicate that global and local 5hmC patterns are robust markers that could distinguish cancer from non-cancer samples, suggesting the potential for 5hmC as a cancer biomarker, although further research is still needed.

## 3. Evolution of Hydroxymethylation Detection Methods

Over the past decade, significant advances have been made for quantifying and profiling the hydroxymethylome. Early 5hmC profiling studies relied on quantification methods, such as mass spectrometry [14,81], thin-layer chromatography [11,82], high-performance liquid chromatography (HPLC) [82,83], immunoassays [53,72], and spectroscopic techniques [84]. While these methods can accurately quantify 5hmC levels, their sample preparation complexity, high equipment and reagent costs, and low resolution make them challenging to implement in the clinical setting. Since then, sequencing-based methods for genome-wide and single-base resolution profiling have been developed [85,86,87,88]. An overview of the most common 5hmC profiling techniques is summarized in Table 1.

### 3.1. Bisulfite Sequencing Approaches

Bisulfite sequencing (BS-seq) is the gold standard approach for DNA methylation profiling, offering genome-wide assessment at a single-base resolution. Traditional BS-seq involves treating DNA with sodium bisulfite, which deaminates unmodified cytosines (C), 5fC, and 5caC to uracil (U) and converts 5hmC to cytosine-5-methylsulfonate (CMS), while leaving 5mC unchanged [89]. Since the final readout of 5mC and CMS are both cytosines, a key limitation of BS-seq is that it cannot distinguish between 5mC and 5hmC [119]. To overcome this barrier, chemicals can be added prior to bisulfite treatment to protect or modify the base, thereby enabling more precise differentiation of methylation states.

Oxidative Bisulfite Sequencing (OxBS-seq) [90] and TET-assisted bisulfite sequencing (TAB-seq) [95] are examples of modified bisulfite techniques. OxBS-seq utilizes potassium perruthenate (KRuO_4_) to oxidize 5hmC into 5fC [90]. Since 5fC is an unstable intermediate, TDG and BER machinery will convert it back to cytosine, allowing it to undergo deamination upon bisulfite treatment [90]. Comparison of readouts against traditional bisulfite sequencing allows for the identification of 5hmC (“C” from BS-seq and “T” from OxBS-seq). TAB-seq, on the other hand, relies on T4 phage β-glucosyltransferase (T4-βGT) to transfer a glucose moiety onto 5hmC, protecting it from oxidation by TET1 [95]. After oxidization and bisulfite treatment, the final readout allows for direct identification of 5hmC (5hmC reads as “C”, while cytosine, 5mC, and other oxidized derivatives are read as “T”).

Although OxBS-seq and TAB-seq offer genome-wide profiling at a single-base resolution and can be leveraged to study the interplay between 5mC and 5hmC, these techniques have several limitations [77,120]. Firstly, they require large amounts of input DNA (>100 ng), posing a challenge for cfDNA or early cancer detection applications [94,121,122]. Secondly, these assays demand a high sequencing depth (>100× coverage), which is expensive and constrains practicality [94,121]. Additionally, since OxBS-seq is usually paired with BS-seq for 5hmC identification, this approach increases costs and the potential for errors when comparing final readouts [90]. Third, bisulfite treatment uses harsh chemicals and carries the risk of DNA degradation [123]. Enhancements to oxBS-seq and TAB-seq, such as integrating of methylation microarrays [91,94,96], using different catalytic enzymes [92,93], and focusing on CpG sites [94,97], have been shown to improve assay sensitivity, minimize DNA damage, and reduce sequencing costs. However, 5hmC regions not included in the provided microarray may be missed and damage from bisulfite treatment remains a concern.

### 3.2. Enzymatic and Affinity-Based Approaches

To overcome the limitations of bisulfite-based methods, bisulfite-free techniques use chemicals, enzymes, and antibodies to glucosylate, deaminate, oxidize, or selectively pull down 5hmC to capture its genome-wide distribution. These strategies, in turn, improve the sensitivity, specificity, and feasibility of 5hmC profiling techniques, easing the transition for clinical utility.

#### 3.2.1. Chemical Capture and Glucosylation-Based Techniques

Glucosylation involves the transfer of a glucose moiety onto the hydroxyl group on 5hmC via T4-βGT to form glucosyl-5hmC (5ghmC). The glucose molecule can be radiolabelled for quantification [124,125], used to protect 5hmC from oxidation [98] or employed to attach various tags onto 5hmC [44,54,99,108,109]. For instance, Glucosylation, Periodate Oxidation, Biotinylation (GLIB) uses glucosylation to protect 5hmC prior to oxidation by sodium periodate (NaIO_4_) [40,98]. This is followed by biotinylation and capture by streptavidin beads. GLIB has several limitations including high background noise caused by sodium periodate oxidation and DNA fragmentation, and slight inhibitory effects on PCR [98]. These may compromise subsequent analyses or underestimate 5hmC-rich regions during sequencing. Though these limitations can be addressed with the assay’s intended negative control (condition without T4-βGT) and replacing PCR with a Helicose instrument, these solutions may incur additional costs and may not be readily available in all laboratories [98].

5hmC-Selective Chemical Labeling (HMe-SEAL) is another glucosylation-based technique that utilizes T4-βGT to transfer a modified azide-glucose molecule onto 5hmC [54]. This is followed by the addition of a biotin tag via click chemistry, and subsequent streptavidin bead pull-down of biotinylated fragments [54]. Currently, optimized versions of HMe-SEAL are widely used for profiling 5hmC from rare cell populations (Nano-hmC-Seal) [44,66,78] and cell-free DNA [58,76,79,126]. However, like other enrichment techniques, HMe-SEAL does not permit single-base resolution of 5hmC sites, limiting its ability to quantify absolute 5hmC levels. Nevertheless, HMe-SEAL presents an attractive opportunity for cfDNA studies as it requires low DNA input and can efficiently enrich for 5hmC-containing fragments, allowing for more cost-effective sequencing [58].

Similarly, J-Binding Protein 1 sequencing (JBP1-seq) also relies on T4-βGT to glucosylate 5hmC [99,100]. Instead of click chemistry, this technique uses J-binding protein 1, which acts as a natural antibody for glucosylated 5hmC. In one study comparing JBP1-seq to HMe-SEAL and other affinity-based 5hmC profiling methods, JBP1-seq displayed significantly different 5hmC profiles and was unable to correlate chromatin states with 5hmC patterns [127]. Moreover, it may be biased towards transcription start sites, leading to lower enrichment of 5hmC [127]. However, an optimized JBP1-seq workflow has demonstrated improved 5hmC enrichment capabilities, quicker performance (4.5 h), and reduced DNA input requirements [100]. Further validation against other techniques is necessary to fully assess the performance of this optimized workflow.

Other glucosylation methods include Jump-seq [108] and 5hmC Tethered Oligonucleotide-Primed Sequencing (hmTOP-seq) [109]. Both Jump-seq and hmTOP-seq use T4-βGT to attach an azide glucose onto 5hmC, followed by click chemistry tagging with a hairpin or tethered oligonucleotide, and primer extension to locate 5hmC [108,109]. Recently, hmTOP-seq has also been used for detecting 5hmC in cfDNA [128]. Though the study revolved around prenatal testing in maternal blood cfDNA, hmTOP-seq demonstrated 100% accuracy in detecting Down syndrome in fetuses, even at ultra-low sequencing depths. This indicates its potential as a tool for profiling the cell-free hydroxymethylome. However, it should be noted that both Jump-seq and hmTOP-seq rely on indirect methods for quantifying 5hmC, which can introduce biases and artifacts post-sequencing [108,109]. Additionally, Jump-seq tends to have a bias towards regions with high CpG density and may not perform well if 5hmC peaks are too close in proximity [108].

#### 3.2.2. DNA Deamination Methods

AID/APOBEC enzymes present an alternative method for deaminating DNA, bypassing the unstable sulfonated intermediate generated during bisulfite treatment. This reaction uses a zinc cofactor to deaminate cytosines on single-stranded DNA [129]. The first technique to employ this strategy was APOBEC-coupled epigenetic sequencing (ACE-seq) [103,104]. In ACE-seq, 5hmC is first glucosylated by T4-βGT to protect it from subsequent deamination by APOBEC3A. 5mC and other unmodified cytosines, on the other hand, will be deaminated upon APOBEC3A treatment. A major advantage of ACE-seq is that it can achieve single-base resolution mapping of 5hmC with 1000-fold less DNA input than BS-seq [103]. Similarly, Enzymatic Methyl sequencing (EM-seq) follows the same workflow as ACE-seq but employs TET2 to oxidize 5mC and 5hmC prior to glucosylation [105].

Single-Step Deamination sequencing (SSD-seq) is another deamination technique that utilizes a specially engineered protein, eA3A-v10, to deaminate cytosine and 5mC, but not 5hmC [111]. This approach is promising as it eliminates the need for additional glucosylation or oxidation steps prior to deamination. Moreover, deamination by eA3A-v10 uses a gentle deamination reaction, overcoming the limitations of DNA damage by bisulfite treatment [111]. Like ACE-seq, it should be noted that the 5hmC readout may include traces of 5fC and 5caC, which can potentially overestimate 5hmC levels. However, since their prevalence and stability are much lower than 5hmC, it may not significantly impact the final result.

Newer deamination approaches combine deamination with existing profiling techniques to enhance the resolution of genome-wide 5hmC detection. DNA Immunoprecipitation-Coupled Chemical Modification-Assisted Bisulfite Sequencing (DIP-CAB-seq) is a three-step strategy that involves: (1) glucosylation, click chemistry, and biotin-streptavidin interaction, like HMe-SEAL, (2) deamination by APOBEC, and (3) single-stranded sequencing [110]. Although DIP-CAB-Seq is labor-intensive, it demonstrates comparable 5hmC enrichment abilities to HMe-SEAL, while achieving improved, single-base resolution detection [110]. Likewise, Enrichment-Based, Single-Base Resolution 5hmC Sequencing (EBS-seq) follows a similar workflow, with deamination by APOBEC occurring before streptavidin bead pull-down [43]. Even when samples had low 5hmC content, like human cancer cell lines and tissues, 5hmC could be efficiently detected [43].

#### 3.2.3. Oxidation-Based Strategies

Oxidation-based, bisulfite-free approaches are commonly used to convert or protect cytosine modifications prior to chemical treatment. For example, Chemical-Assisted C-to-T Conversion of 5hmC Sequencing (hmC-CATCH) uses potassium perruthenate (KRuO_4_) to oxidize and convert 5hmC to 5fC, prior to labeling with indandione [106]. This process ensures that only 5fC-labelled products undergo a “C-to-T” transition during PCR, while other cytosine derivatives remain unchanged [106]. Another technique, TET-assisted pyridine borane sequencing (TAPS-seq), uses TET to oxidize 5mC and 5hmC to 5caC [107]. Unlike TAB-seq, it uses pyridine borane to reduce 5caC to dihydrouracil (DHU), rather than bisulfite treatment. When performed in parallel to TAPSβ-seq, which incorporates glucosylation to protect 5hmC from oxidation, their collective readouts allow for the identification of 5hmC. Although this assay requires a lengthy incubation time in acidic conditions, it is less destructive than bisulfite treatment and requires low amounts of input DNA to operate, making it suitable for use with cfDNA [107,130].

Oxidation techniques offer several advantages. For instance, hmC-CATCH, compared to TAB-seq, requires less input DNA (100 ng in hmC-CATCH vs. 1 µg in TAB-seq) and sequencing depth, while demonstrating similar 5hmC enrichment patterns in human embryonic stem cells [106]. Moreover, hmC-CATCH is compatible with cfDNA input and displays similar 5hmC enrichment profiles as HMe-SEAL [106]. However, since hmC-CATCH relies heavily on “C-to-T” conversions for 5hmC identification, incomplete conversions may underestimate 5hmC levels. On the contrary, 5hmC levels may be overestimated since endogenous 5fC could also undergo “C-to-T” transitions. However, pre-blocking with O-ethylhydroxylamine (EtONH_2_) prior to 5hmC oxidation can mitigate this limitation [106].

#### 3.2.4. Antibody-Based Methods

Antibodies specific for 5hmC or its derivatives can also be used to capture hydroxymethylated DNA fragments [40,101,102]. Hydroxymethylated DNA Immunoprecipitation (hmeDIP) [101] utilizes monoclonal, anti-5hmC antibodies to capture and pull down hydroxymethylated DNA and has been implemented widely for both local and genome-wide profiling of 5hmC in cancer tissues and cell lines [68,94,131]. Although antibody-based profiling techniques are limited by the variability in antibody binding efficiency and may be biased towards hyper-hydroxymethylated regions, they are cost-effective, easy to implement, and can be highly specific for 5hmC [101].

### 3.3. Emerging Hydroxymethylation Profiling Methods

Single Molecule, Real Time sequencing (SMRT) [112,113] was the first to simultaneously sequence 5hmC and the genome without the need for chemical or bisulfite treatment. In SMRT sequencing, fluorescent pulses are detected as fluorescently labelled nucleotides are incorporated into the primary sequence by DNA polymerase [112]. Modified cytosines can be detected by their unique kinetic signature, though the specificity of these signatures for 5hmC detection remains a challenge [112]. In 2011, Song and colleagues enhanced 5hmC detection by enriching for 5hmC via glucosylation, biotin, and streptavidin bead pull-down, prior to conducting SMRT sequencing [113]. This approach reduced sequencing costs and improved the bulkiness of the adduct, making it easier to distinguish from other cytosine modifications. However, this method’s high reagent costs, large DNA input requirements, and the lack of standardized bioinformatics infrastructure limits its use for 5hmC detection.

Recently, growing enthusiasm for understanding the interplay between epigenetic and genetic alterations has sparked the development of several new simultaneous sequencing techniques. The 6-letter sequencing technique by Füllgrabe et al. (2023) is an approach that expands the genetic alphabet to six bases, rather than the usual four [114]. This technique uses synthetic DNA hairpin adapters to separate and synthesize a complementary strand lacking epigenetic modifications, resulting in a “copied” (5mC) strand and an “uncopied” (5hmC) strand. This is followed by treatment with T4-βGT on the “uncopied” strand, and DNMT5, TET2, UvrD helicase, and APOBEC3A on both strands. After paired-end sequencing, two-base resolutions rules are applied to deconvolute pairwise alignments. This method offers a promising solution to previous simultaneous sequencing efforts as it requires low amounts of input DNA, making it suitable for cell-free DNA applications [114]. Other simultaneous sequencing technology like optical imaging [132] and nanopore sequencing [133] also provide opportunities for direct, simultaneous sequencing of the epigenome and genome, as summarized in previous reviews [134,135].

Restriction enzymes can also be used for simultaneous sequencing. DNA Analysis By Restriction Enzyme For Simultaneous Detection of Multiple Epigenomic States (DARESOME) utilizes a series of digestion (by restriction enzymes Hpa II and Msp I), adaptor tagging (with U-tag, H-tag, and M-tag) and glucosylation steps to simultaneously profile the methylome and hydroxymethylome [115]. DARESOME features a single-tube workflow and is compatible with low amounts of input DNA, making it suitable for cfDNA or single cells [115]. Dyad-seq is another restriction enzyme-based method designed to quantify 5mC and 5hmC at all CpG dyad combinations on both strands of DNA (H-H, M-H, M-M, and H-M) [118]. This method involves fours steps: (1) glucosylation by T4-βGT, (2) enzymatic digestion by MspJI (for detecting 5mC on the top strand) or AbaSI (for detecting glucosylated 5hmC on the top strand), (3) adaptor ligation with a random overhang, and (4) enzymatic processing to identify modifications on the bottom strand. For 5hmC detection, 5hmC is glucosylated by T4-βGT, followed by deamination by APOBEC3A. For 5mC detection, it involves oxidation by TET2, glucosylation by T4-βGT, and deamination by either APOBEC3A or sodium bisulfite treatment. One key advantage of this assay is its opportunity to integrate epigenomic and single-cell transcriptomic data (scDyad&T-seq) [118]. While the authors have only attempted this for methylation profiling (M-M-dyad-seq), future extension to profiling the hydroxymethylome will make this assay attractive for multi-omics analyses [118].

5hmC single-cell sequencing techniques can be used to shed light on the complexity and heterogeneity of a sample by studying the dynamics and cellular diversity of 5hmC within individual cells. For example, Single-Cell Intracellular Metabolite Profiling and Labeling Experiment Sequencing (SIMPLE-seq) is a technique that integrates hmC-CATCH and TAPS sequentially to detect 5hmC at the single-cell level [116]. It relies on a series of oxidation, reduction, and primer tagging steps to form and record two “C-to-T” conversions within the same mixture. Using SIMPLE-seq on a seemingly homogenous population of mouse embryonic stem cells, Bai and colleagues (2024) observed distinct 5mC and 5hmC profiles in different cells [116]. They also demonstrated that 5mC and 5hmC could distinguish different cell populations, highlighting the versatility of SIMPLE-seq and value of simultaneously sequencing 5mC and 5hmC in a single-cell context [116]. Besides SIMPLE-seq, other recently developed single-cell 5hmC techniques include Carrier-Assisted Base-conversion by Enzymatic ReactioN with End-Tagging (Cabernet) [136], single-cell DARESOME (scDARESOME) [115], and joint-snhmC-seq [117].

Despite the comprehensive insights provided by simultaneously sequencing the epigenome and genome in a single workflow, current methods have several limitations. For example, the 6-letter sequencing technique can only distinguish between 5mC from 5hmC at CpG regions and is relatively expensive due to the need for high depth, paired-end sequencing [114]. Similarly, DARESOME only operates at CCGG sites, which represents only 10% of CG sites in the genome [115]. Additionally, analysis for restriction enzyme-based approaches, like DARESOME and Dyad-seq, is limited to only one site per DNA fragment, restricting the ability to analyze neighbouring 5hmC and 5mC sites [118]. Furthermore, since SIMPLE-seq relies on “C-to-T” conversions, improper conversion or mislabeling of endogenous 5fC may result in false positive readings [116]. Addressing these limitations will be essential for increasing the use of these assays in the future.

## 4. DNA Hydroxymethylation and Clinical Applications

**Figure 3:**
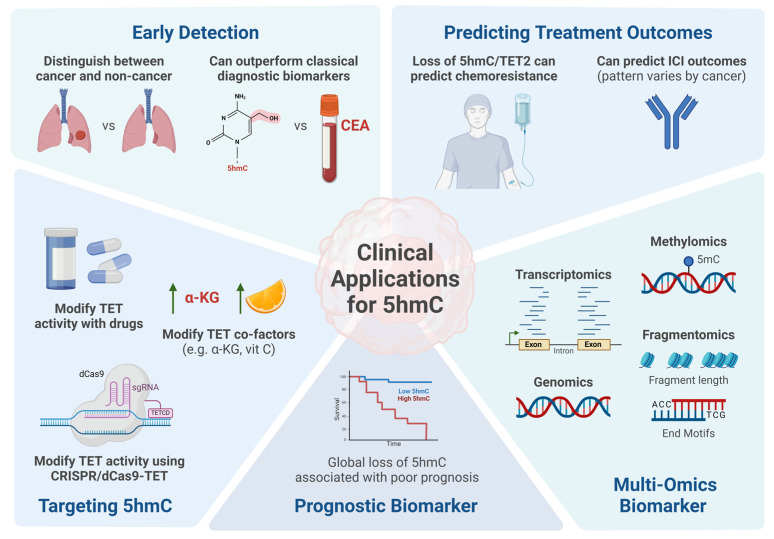
Clinical Applications for DNA Hydroxymethylation in Cancer. DNA hydroxymethylation (5hmC) has been leveraged as a biomarker for early detection, predicting treatment outcomes, and disease prognosis in multiple cancers. It can also be integrated with other omics datasets, such as methylomics, transcriptomics, fragmentomics, and genomics, to provide a more comprehensive understanding of the biology underlying tumorigenesis. Moreover, studies have explored the effects of modifying TET activity or co-factors to alter 5hmC levels as a potential therapeutic option for cancer. CEA, carcinoembryonic antigen; TET, ten-eleven translocation dioxygenase; ICI, immune checkpoint inhibitor; α-KG, alpha ketoglutarate; vit C, vitamin C; dCas9, deactivated Cas9; sgRNA, single guide RNA; TETCD, TET catalytic domain; 5mC, 5-methylcytosine/DNA methylation. This figure was created with Biorender.com.

5hmC has emerged as a promising biomarker for cancer, displaying distinct global and local patterns that can differentiate between cancerous and non-cancerous samples. Previous studies have explored its potential as a diagnostic, prognostic, and predictive biomarker for cancer, underscoring its utility in cancer management. Figure 3 and Table 2 highlight examples of clinical applications of 5hmC as a cancer biomarker.

### 4.1. DNA Hydroxymethylation as a Biomarker for Cancer Detection

Early detection of cancer enables more effective treatment and better outcomes, making it crucial to have reliable diagnostic biomarkers. However, this is challenging since tumour content is imperceptibly low at early stages, especially ctDNA [146]. Global and locus-specific 5hmC patterns are promising diagnostic biomarkers as these epigenomic changes occur early in tumorigenesis and can distinguish between cancerous and non-cancerous states [58,59,60,61,62,76,78,80,137,147].

In a pan-cancer study, Shao et al. (2022) established a 24-5hmC-gene model using cfDNA from healthy individuals and patients with bladder, breast, colorectal, kidney, lung, or prostate cancer [78]. Their model could successfully distinguish between cancer and healthy individuals with high performance in their validation cohort (area under the curve [AUC] = 91%, sensitivity = 68.6%, and specificity = 96.6%). When considering each cancer individually, their cancer-specific weighted diagnostic (wd) models achieved AUCs ranging from 94–99.8%, sensitivities ranging from 80–96%, and specificities ranging from 96–100%. Performance was also high for detecting early-stage disease, with pan-cancer sensitivities at 71.4% and 81.3% and cancer-specific signatures at 89.3% and 94.1% for stage I and II disease, respectively.

In a multicenter study, METHOD-2 (NCT03676075), Chang et al. (2024) also developed a cfDNA 5hmC classifier capable of distinguishing between stage I-III colorectal cancer patients and non-cancer controls [137]. Their model based on 96 5hmC gene bodies achieved an AUC of 94.3% in internal validation and 90.7% in external validation. Notably, their cfDNA 5hmC-based classifier outperformed the detection performance of a classical colorectal biomarker, carcinoembryonic antigen (CEA), which had an AUC of 77.1% and 73.2% in the internal and external validation cohorts, respectively. At 90% specificity, the 5hmC-based score achieved sensitivities of 73.5% for Stage I and 85.3% for Stage II/III colorectal cancer, whereas CEA achieved sensitivities of 29.4% and 47.2% for the same stages. Similarly, studies in hepatocellular carcinoma [61], lung cancer [60,76], and colorectal cancer [59] have also shown that 5hmC-based models could outperform conventional diagnostic biomarkers, highlighting the potential of 5hmC in cancer detection.

Though global and gene-specific cell-free 5hmC patterns hold potential as diagnostic biomarkers for cancer, even for early-stage disease, several gaps remain to be addressed before these models can be translated into clinical practice. Firstly, current diagnostic models tend to focus solely on gene-specific 5hmC signatures, whereas other 5hmC-enriched regulatory elements, such as enhancers, are often overlooked. Incorporating other elements into these models could enhance the performance and improve our understanding of biology behind observed 5hmC patterns. Secondly, future studies should prioritize validating existing gene signature models, particularly in different geographical and ethnic populations, to assess the generalizability of these models.

### 4.2. Prognostic Value of DNA Hydroxymethylation in Cancer

Beyond early detection, there is evidence supporting the value of 5hmC profiling in cancer prognostication [56,63,64,65,69,72,73,74,75,138,139,140,148,149,150,151]. For instance, in intrahepatic cholangiocarcinoma (ICC), Dong et al. (2015) showed that low or negative 5hmC levels in tissue were associated with a higher tumour stage, more lymph node metastases, shorter overall survival, and worse disease-free survival compared to high/positive 5hmC levels [72]. Likewise, Kuang and colleagues (2024) revealed that *TET2*^−^/5hmC^−^ endometrial adenocarcinoma was associated with the poorest overall survival (*p* < 0.001) amongst other combinations of *TET2* and 5hmC expression [139]. In contrast, *TET2*^+^/5hmC^+^ endometrial cancer was significantly associated with well-differentiated cells, minimal myometrial invasion, negative lymph node metastasis, and lower tumour stage [139]. Multivariate analysis further demonstrated that the *TET2*/5hmC relationship could be an independent prognostic factor for endometrial cancer (HR: 2.84, 95% confidence interval [CI] 1.23–3.61, *p* = 0.007). Similarly, in gastric cancer, Fu et al. (2022) demonstrated that high 5hmC was an independent, favourable predictor of OS (hazard ratio [HR] = 0.61, 95% CI 0.38–0.98, *p* = 0.04) and lower tumour stage (HR = 0.32, 95% CI 0.13–0.77, *p* = 0.011) [138].

Cell-free 5hmC signatures could also be used for cancer prognosis or patient risk stratification [63,64,65,79,140]. In diffuse large B-cell lymphoma (DLBCL), Chiu et al. (2019) created a 29 5hmC gene-based weighted prognostic (wp) model that achieved 96% accuracy, 86% sensitivity, and 100% specificity for predicting event-free survival (EFS) and OS [63]. Patients with a high weighted prognostic (wp-score) had significantly worse OS and EFS, compared to those with a low wp-score. Notably, sensitivity, specificity, and accuracy of the 5hmC model outperformed existing DLBCL prognostic factors, such as elevated levels of lactate dehydrogenase, activated B-cell-type DLCBL, and high international prognostic index (accuracy = 36–81%, sensitivity = 56–80%, specificity = 29–89%).

In hepatocellular carcinoma (HCC), Cai et al. (2021) developed an HCC score using a cfDNA 5hmC signature and two HCC protein biomarkers (alpha-fetoprotein [AFP] and des-gamma-carboxy prothrombin [DCP], Table 2) [64]. A high HCC score was positively correlated with clinicopathological prognostic factors of HCC, such as microvascular invasion, tumour size, and tumour stage, and was significantly associated with greater recurrence rates, shorter relapse-free survival, and shorter OS. Interestingly, the HCC score was also positively associated with real-time tumour burden dynamics, suggesting its potential for predicting disease recurrence. However, this finding was limited by the small sample of the study.

In another study, Shao et al. (2023) stratified 54 acute myeloid leukemia (AML) patients into three clusters using unsupervised hierarchical clustering on the top 500 most variable cfDNA 5hmC regions marked by H3K4me3 [65]. Cluster 3 exhibited the lowest 5hmC levels in the *TET2* promoter, the shortest OS, and highest leukemia burden. Differentially hydroxymethylated regions (DhMR) between Cluster 3 and Cluster 1 were enriched in cell survival/proliferation pathways (i.e., mTOR, ERK/MAPK, and insulin receptor signalling pathways) which are associated with poor prognosis.

Similarly, in NSCLC, Shao and colleagues (2024) created a prognostic model using a 17 5hmC-gene signature [140]. Patients with a low wp-score were associated with significantly longer median OS (18.8 vs. 5.2 months, *p* = 0.0006; HR 0.22, 95% CI 0.09–0.57) and median PFS (8.8 vs. 3.3 months, *p* = 0.054; HR 0.45; 95% CI 0.20–1.00), compared to those with a high wp-score. The prognostic score was more accurate in predicting survival outcomes than well-established clinical factors, like age, sex, smoking history, and tumour stage.

Collectively, these studies indicate that cell-free and tissue-based 5hmC patterns could serve as a prognostic biomarker in cancer. Further investigation into the genes and pathways underlying these 5hmC patterns will offer deeper insights into the factors contributing to the risk of poor prognosis.

### 4.3. Predicting Chemotherapy and Immunotherapy Response with 5hmC

Alterations in 5hmC patterns can be leveraged to predict patient response to cancer therapeutics. Recent studies have revealed a potential relationship between 5hmC expression levels and chemotherapy response [141,142]. In DLCBL, Chen et al. (2021) demonstrated that responders and non-responders to the chemotherapy regimen R-CHOP (rituximab, cyclophosphamide, doxorubicin, vincristine, and prednisone) had distinct pre-treatment 5hmC profiles [142]. Their 13 cfDNA 5hmC logistic model achieved an AUC of 78%, sensitivity of 82% and specificity of 75% in the validation cohort, demonstrating 5hmC could distinguish between responders and non-responders. In hepatocellular carcinoma (HCC), Guo and colleagues (2023) demonstrated that the loss of 5hmC induced chemoresistance through the histone acetyltransferase P300/CBP-associated factor (PCAF)/AKT axis [141]. The authors revealed that chemoresistant HCC tissue and cell lines had significant reductions in global 5hmC and *TET2* expression levels and correlated with poor cell differentiation and increased microvascular invasion. Upon exogenous overexpression of *TET2*, global 5hmC levels were restored and cells were resensitized to chemotherapy. In contrast, knockdown of *TET2* in chemosensitive cells reduced global 5hmC levels, decreased PCAF expression, and increased AKT signalling, which are pathways associated with chemoresistance. Furthermore, *TET2* knockdown inhibited apoptosis and promoted cell proliferation, suggesting that reduced TET2 activity and subsequent loss of 5hmC promotes chemoresistance [141].

5hmC and dysregulation in TET enzymes have also been implicated in the regulation of key immune genes involved in T-cell differentiation, activation, and exhaustion, suggesting its potential for predicting immune checkpoint inhibitor (ICI) response [32,33,34,35]. Wu and colleagues showed that mutated *TET1* is associated with significantly longer overall survival (OS), better objective response rate (ORR), improved durable clinical benefit (DCB), and better progression-free survival (PFS) in cancer patients receiving ICI [152]. Interestingly, other studies in renal cell carcinoma and melanoma observed the opposite effect, in which increasing TET-dependent demethylation substrates, like vitamin C and α-KG, improved *TET2* activity at the promoter of interferon regulatory factor 1 (IRF1) or programmed death-ligand 1 (PD-L1), leading to their demethylation and downstream increase in PD-L1 expression [153,154]. This, in turn, promoted intratumoral T-cells infiltration and ICI efficacy [153,154].

Recently, Shao et al. (2024) developed a predictive model using a 16-gene cell-free 5hmC signature that could predict ICI outcomes in late-stage non-small cell lung cancer (NSCLC) patients [66]. Patients with a low weighted predictive score were significantly associated with longer median OS and better objective response rates [66]. Their cell-free 5hmC signature also demonstrated superior predictive capability than PD-L1 tumour proportion score (TPS), a widely used predictor for ICI decisions [66,155]. Guler and colleagues (2024) also investigated the dynamics of cell-free 5hmC in NSCLC and observed that ICI responders and non-responders had widely different 5hmC patterns [143]. Responders to ICI had 5hmC gains in immune cell activation genes (i.e., *HLA-DQB1* and *CD69*) and 5hmC loss at cancer or drug-resistant genes (i.e., *IGF1*, *TWIST1*, and *MMP16*), whereas ICI non-responders had 5hmC enrichment in genes associated with ICI resistance (i.e., *FGF*) and 5hmC depletion in immune function genes (e.g., *CD74*). Though further studies are required to fully understand the role of 5hmC in ICI treatment, cfDNA 5hmC shows promise as a potential predictor for ICI response.

### 4.4. Integrating DNA Hydroxymethylation in Multi-Omics Analysis

As cancer research is expanding, it is becoming more evident that cancer is the sum of several underlying biological networks, rather than just one phenomenon in isolation [156]. As such, there is growing interest in studying the integration and interplay of DNA 5hmC with other layers of biology, such as methylomics, fragmentomics, and transcriptomics. In particular, studying cfDNA fragment features (fragmentomics), like size, end motifs, jagged ends, nucleosome positioning, and coverage, can reveal differences between tumour and non-tumour DNA [157]. For example, ctDNA fragment lengths are typically shorter than cfDNA fragments (~145 base pairs [bp] vs. ~166bp in cfDNA) [158]. In NSCLC, Hu and colleagues (2022) developed an integrated 5hmC and fragmentomics model for early detection [126]. Using 37 cfDNA 5hmC markers and 48 fragmentation features (ratio of short-to-long fragments at different windows), their model distinguished NSCLC patients from non-cancer controls and achieved AUCs of 86–94%, sensitivities of 83–88%, and specificities of 78–90% in two validation cohorts. Moreover, this integrated model outperformed standalone 5hmC and fragmentation models, highlighting the value in combining fragmentomics lengths with cfDNA 5hmC for cancer detection. In a pan-cancer study, Zhang et al. (2023) established a diagnostic model using 5hmC signatures and multiple cfDNA fragment features, including size, coverage, and preferred ends [144]. Their model achieved high AUC values, sensitivity, and specificity, demonstrating the potential of integrated 5hmC and fragment features for cancer diagnosis [144]. Integrated 5hmC and fragmentomics models have also been studied for early detection in other cancers [159,160].

Previous groups have also explored the interplay between 5mC, 5hmC, and gene expression to gain a deeper understanding of the genetic and epigenetic patterns underlying cancer [47,79,115,117,145,161,162]. Shi et al. (2023) combined data from the genome, transcriptome, methylome, and hydroxymethylome to better understand the development of bladder cancer relapse [145]. They demonstrated that despite the absence of known driver mutations for bladder cancer, 5hmC- or 5mC-induced transcription alterations were linked to pathways associated with bladder cancer recurrence and tumour immune escape, respectively. This suggests that epigenetic alterations may play a more significant role in bladder cancer recurrence than genomic mutations [145]. In pediatric central nervous system (CNS) cancers, Lee et al. (2024) integrated 5mC, 5hmC and single-nucleus RNA-seq data to investigate the impact of cell-type composition on epigenetics [47]. The authors found that 5mC and 5hmC abundance and alterations were highly influenced by the composition and heterogeneity of the tumour. Interestingly, they also observed similar prevalence in epigenome-wide dysregulation between 5mC and 5hmC in tumour tissue, despite significantly lower abundance of 5hmC compared to 5mC in the genome, indicating the independent and crucial role of 5hmC in tumorigenesis [47].

Therefore, the integration of 5hmC with other omics datasets offers opportunities to enhance current cancer biomarkers and deepens the understanding of 5hmC patterns and cancer pathophysiology. Challenges and future directions are discussed further below in Section 5.2.

### 4.5. Targeting DNA Hydroxymethylation as a Potential Therapeutic for Cancer

While many have reported on global and local patterns of 5hmC in cancer, the specific mechanisms driving these changes remain elusive. Reduction in 5hmC levels has been associated with impaired TET activity, which can result from inactivating mutations in TET genes [163,164,165,166,167], downregulation of TET expression [74,139,168,169], and inhibition of TET co-factors and substrates [170]. Previous studies have shown that overexpressing TET could significantly inhibit cell proliferation, migration, and invasion, whereas knocking down TET exhibited the opposite phenotype [75,131,141,169]. Therefore, targeting TET may offer a potential strategy for cancer treatment.

To our knowledge, there are currently no approved drugs that specifically target TET proteins for cancer treatment. However, previous drug screening studies in AML have revealed compounds, such as NSC-311068 and NSC-370284, that could suppress *TET1* expression and, subsequently, 5hmC levels, to inhibit cell viability and tumour progression in vitro and in vivo [171]. Another emerging strategy involves the use of CRISPR/cas9 epigenome editing tools, in which a deactivated cas9 (dcas9) is fused to the catalytic domain of a TET protein (TETCD) to target and demethylate DNA at genic regions specified by the guiding RNAs (sgRNA) [172,173,174,175]. Preliminary works by Choudhury et al. (2016) using a CRISPR/dCas9-TET1CD system demonstrated successful demethylation of the *BRCA1* promoter in vitro [172]. This, in turn, rescued *BRCA1* expression and inhibited cell growth in multiple cancer cell lines. Similarly, Xu et al. (2018) used a high-fidelity dcas9 coupled to TET3CD to demethylate hypermethylated anti-fibrotic genes in kidney fibrosis [174]. Here, they demonstrated successful gene-specific reactivation of anti-fibrotic genes, *Rasal1* and *Klotho,* in vitro and in vivo. Future directions such as optimizing the sgRNA binding sites relative to the target and investigating off-target effects will improve the extent and utility of this CRISPR/Cas9 technology.

TET can also be indirectly modified by altering the availability of its substrates and co-factors. For instance, alpha ketoglutarate (α-KG) is a key intermediate in the tricarboxylic acid cycle (TCA) that also serves as a substrate of TET in the demethylation pathway [176]. A study by Liu et al. (2023) demonstrated that α-KG supplementation increased TET2/3 activity in melanoma, resulting in elevated 5hmC levels at the PD-L1 promoter and improved ICI efficacy [154]. Mutations in isocitrate dehydrogenase (IDH) genes *IDH1* and *IDH2* may also impact TET activity as the mutant enzyme converts α-KG into 2-hydroxyglutarate, a competitive inhibitor for the TET catalytic site [177,178]. However, it remains uncertain whether mutations in IDH are the primary cause of 5hmC depletion in cancer [179].

Vitamin C is another cofactor that can enhance TET activity, thereby increasing 5hmC levels [180,181]. Peng et al. (2018) demonstrated that vitamin C treatment could restore 5hmC levels in a time- and concentration-dependent manner while suppressing cell proliferation and inducing apoptosis in bladder and renal cancer cell lines [68]. Interestingly, despite increased TET activity, *TET1/2/3* expression levels did not change significantly, indicating that vitamin C enhances TET activity, rather than expression level [68]. Similar findings have also been observed in other cancers [75,182,183], suggesting that vitamin C treatment could be a potential therapeutic option for cancer.

Although both α-KG and vitamin C show potential in altering TET activity, these co-factors are also involved in multiple cellular processes and as such can lead to unintended side effects [170]. While these preclinical studies are promising, further investigation into the exact mechanisms and consequences of altering these co-factors is necessary.

## 5. Challenges and Future Directions

### 5.1. Choosing the Right 5hmC Detection Method

Advancements in 5hmC detection methods have led to improved genome-wide profiling strategies for studying the hydroxymethylome. Current techniques can now differentiate between various cytosine modifications (Table 1), allowing for the individual evaluation of each modification’s role in tumorigenesis. Novel approaches that simultaneously sequence the genome and epigenome hold great promise, offering opportunities for multi-omics analyses using a single workflow [114,115,116,117,118,136]. However, challenges such as the DNA input requirements and sequencing costs persist. Moreover, as the use of cfDNA inputs continues to grow, it is essential to have sensitive assays capable of detecting 5hmC, even at low concentrations. Nonetheless, developments in liquid biopsy-based techniques are emerging and can enhance the sensitivity and specificity of cfDNA detection. The decision to choose one method over the other may be influenced by how well the assay addresses these barriers or the availability of the reagents required by the method of choice.

### 5.2. Multi-Omics Analyses Using Cell-Free DNA Hydroxymethylation

Profiling the cell-free hydroxymethylome for cancer detection, prognosis, and predicting treatment outcomes is gaining traction. Integrating 5hmC data with other omics will not only complement current biomarker models [126,144,159,160] but provide a more comprehensive understanding of cancer biology [47,79,115,117,145,161,162]. However, several challenges need to be addressed before clinical implementation is feasible. Firstly, the limited amount of ctDNA in each plasma sample (5–10 ng/mL) poses a challenge for multi-omics studies as there is often inadequate amounts of ctDNA for multiple assays [184]. This makes techniques like 6-letter sequencing [114] and SIMPLE-seq [116] attractive, as they can study the epigenome and genome simultaneously in a single assay while avoiding the DNA damage and fragmentation caused by bisulfite techniques. Additionally, current methodologies have limited sensitivity to detect ctDNA in asymptomatic cancers, hindering early detection efforts [185]. Improving the sensitivity of current assays to detect low amounts of ctDNA will be favourable. Secondly, standardization methods for multi-omics cfDNA studies are still lacking as this is still an evolving field. The development of open-source bioinformatics pipelines, ctDNA deconvolution methods, and synthetic reference controls (i.e., spike-in DNA) will help ensure the reproducibility and comparability of integrated data. Thirdly, epigenetic signals from the cfDNA in the tumour microenvironment or peripheral blood leukocytes may disrupt or dilute the ctDNA signal being studied [186,187]. Therefore, it is crucial to have the proper tools for refining the tumour-specific 5hmC signal, while filtering out background, non-tumour noise. Current tools include variant calling for ctDNA [188] and filtering out peripheral blood leukocyte signals [189], but future ones adaptable to 5hmC ctDNA will be beneficial. Finally, having the proper bioinformatics tools and infrastructure is essential to support integration and analysis of new findings.

## 6. Conclusions

In summary, DNA hydroxymethylation holds great promise as a biomarker for cancer detection, prognosis, and therapeutic response prediction. Advances in profiling methods, particularly in cfDNA approaches, have enabled the development of highly sensitive and specific tools for interrogating the hydroxymethylome. These developments are further enhanced by integrating 5hmC data with other multi-omics datasets, providing a more comprehensive view of cancer mechanisms and aiding in the discovery of novel biomarkers and therapeutic targets. However, several challenges remain to be addressed before broader clinical adoption of 5hmC biomarkers. These include further validating current biomarkers, addressing sequencing and input-related obstacles, and establishing the necessary bioinformatics tools and infrastructure for analysis. Nevertheless, with continued research into 5hmC mechanisms and detection methods, 5hmC has the potential to become a valuable biomarker for improving cancer care.

## Figures and Tables

**Table 1:** DNA Hydroxymethylation Sequencing-Based Detection Methods.

Method	Year	Description	Advantages	Disadvantages
Enzymatic/bisulfite (BS) sequencing methods				
BS-seq [89] (Bisulfite sequencing)	1992	- Sodium bisulfite deamination - * Final readout: T, C, C, T, T	- Gold standard - Single-base resolution	- DNA degradation - Can’t distinguish between 5mC/5hmC - Requires large amounts of input DNA - High sequencing depth
OxBS-seq [90] (Oxidative BS-seq)	2012	- KRuO_4_ oxidation, followed by BS-seq - Results are compared with BS-seq to identify 5hmC - * Final readout: T, C, T, T, T - Similar assays: OxBS-450K (OxBS-seq + Infinium 459K BeadChips) [91], OxBS-seq with catalytic enzymes [92,93], RRBS oxBS-seq (reduced representation oxBS-seq) [94]	- Single-base resolution - Doesn’t require specialized enzymes - Can distinguish between 5mC and 5hmC (indirectly)	- Requires parallel BS-seq - Multiple bisulfite treatments - Increased error rate and costs when comparing between the two methods - Requires large amounts of input DNA - High sequencing depth - Only compares 5hmC at CpG sites - DNA degradation
TAB-seq [95] (TET-assisted BS-seq)	2012	- T4-βGT glucosylation, followed by TET oxidation and BS-seq - * Final readout: T, T, C, T, T - Similar assays: TAB-EPIC (TAB-seq + Infinium methylation EPIC BeadChip array) [96], RRBS TAB-seq (reduced representation TAB-seq) [97]	- Single-base resolution - Can distinguish between 5mC and 5hmC (directly)	- DNA degradation - TET enzymes may self-inactivate - Requires large amounts of input DNA - High sequencing depth
Enzymatic or affinity-based methods				
GLIB [40,98] (Glucosylation, periodate oxidation, biotinylation)	2011	- T4-βGT glucosylation of 5hmC, followed by oxidation by NaIO_4_, biotin tagging, and streptavidin bead pull-down of biotinylated DNA - Final readout: 5hmC is enriched	- Highly specific biotin-based pull-down - Materials are easily accessible - Built-in negative control	- High background noise from periodate oxidation and DNA fragmentation - Slight PCR inhibition
HMe-SEAL [54,58] (5hmC-selective chemical labeling assay)	2011	- T4-βGT attaches azide glucose onto 5hmC, followed by biotin tagging via click chemistry, and streptavidin bead pull-down - Final readout: 5hmC is enriched - Similar assay: Nano-hmC-SEAL [44]	- Highly specific, click chemistry-based pull-down - Genome-wide profiling - Cost-effective - Low DNA input requirement - Can use cfDNA as input	- Multiple steps of chemical labeling - Not single-base resolution
JBP1-seq [99,100] (J-binding protein 1 sequencing)	2012	- T4-βGT glucosylation of 5hmC, followed by pull down using JBP-1 magnetic beads	- Time efficient - Low DNA input requirement - Cost-effective	- Bias towards enrichment at transcription start sites - Limited validation in other studies
hMeDIP-seq [101] (5hmC DNA immunoprecipitation)	2014	- 5hmC-specific antibody used to immunoprecipitate 5hmC - Final readout: 5hmC is enriched - Other uses of antibodies: antibodies against CMS [40], ELISA with anti-5hmC antibodies [102]	- Highly specific, antibody-based pull-down - Genome-wide profiling - Cost-effective - Specific for 5hmC (can’t detect other cytosine modifications)	- Depends on antibody quality (non-specific binding, varying antibody specificity) - Bias towards hypermethylated regions - Not single-base resolution
ACE-seq [103,104] (APOBEC-coupled epigenetic sequencing)	2018	- T4-βGT glucosylation of 5hmC, then deamination by APOBEC3A - * Final readout: T, T, C, C, C - Similar assay: EM-seq (enzymatic methyl sequencing) [105]	- Single-base resolution - Low DNA input requirement	- Has sequence preferences - Time-consuming and costly - May overestimate 5hmC if 5fC and 5caC are detected
hmC-CATCH [106] (Chemical-assisted C-to-T conversion of 5hmC sequencing)	2018	- K_2_RuO_4_ oxidation of 5hmC, then labeling by indandione - * Final readout: C, C, T, C, C	- Single-base resolution - Highly selective for newly generated 5fC (derived from 5hmC) and not endogenous 5fC - Low DNA input requirements - Can use cfDNA as input	- Potential incomplete conversion, leading to underestimation of 5hmC - Complex, multi-step assay
TAPS-seq [107] (TET-assisted pyridine borane sequencing)	2019	- TET oxidation of 5mC and 5hmC, followed by pyridine borane to reduce them to DHU. - Compare outputs with TAPSβ-seq (glucosylation of 5hmC, then oxidation) - * Final readout (TAPS-seq): C, T, T, T, T - * Final readout (TAPSβ-seq): C, T, C, T, T - Similar assay: CAPS (chemical-assisted pyridine borane sequencing) [107]	- Doesn’t degrade DNA - High mapping rate - Cost-effective - Can use cfDNA as input	- Labour-intensive and time-consuming - Low resolution
Jump-seq [108]	2019	- T4-βGT attaches azide glucose onto 5hmC, then labeling with a hairpin oligonucleotide. Primer extension from the hairpin to the 5hmC site allows for 5hmC localization	- Allows for locus-specific quantification, depending on the primer used - Cost-effective	- Bias for high CpG density - Limited testing in clinical samples - Indirect sequence readout
hmTOP-seq [109] (5hmC-specific tethered oligonucleotide-primed sequencing)	2020	- T4-βGT attaches azide glucose onto 5hmC, followed by labeling with an oligonucleotide containing biotin. After streptavidin bead capture, the tethered oligonucleotide-primed strand is amplified and sequenced	- Single-base resolution - Strand-specific 5hmC detection - Low DNA input requirement - Cost-effective - Specific for 5hmC (can’t detect other cytosine modifications) - Can use cfDNA as input	- Affected by genomic variations (i.e., copy number variants) - Does not directly quantify 5hmC (infers 5hmC levels based on sequencing coverage)
DIP-CAB-Seq [110] (DNA immunoprecipitation-coupled chemical modification-assisted bisulfite sequencing)	2021	- 3 steps: ○ (1) T4-βGT glucosylation of 5hmC, click chemistry biotin tagging, streptavidin bead pull-down ○ (2) Deamination by APOBEC ○ (3) Single-stranded sequencing - * Final readout: T, T, C, C, C	- Single-base resolution - Bisulfite-free sequencing - Highly sensitive due to enrichment step prior to chemical conversion - Specific for 5hmC (can’t detect other cytosine modifications)	- Labour-intensive and time-consuming - Does not directly quantify 5hmC - Depends on antibody quality
SSD-seq [111] (Single-step deamination sequencing)	2023	- eA3A-v10 (engineered protein) deaminates C and 5mC, but not 5hmC - * Final readout: T, T, C, C, C	- Single-base resolution - Cost-effective - Straightforward experimental and analytical process - Gentle deamination reaction - No sequence bias - Can use cfDNA as input	- 5hmC readout may include 5fC and 5caC, resulting in potential false positive results - eA3A-v10 requires specialized production and may limit accessibility of the assay
EBS-seq [43] (Enrichment-based sequencing)	2023	- 2 steps: ○ (1) T4-βGT glucosylation of 5hmC, click chemistry biotin tagging ○ (2) APOBEC deamination of 5mC, streptavidin bead pull-down - * Final readout: T, T, C, T, T	- Enrichment method with single-base resolution - Cost-effective	- Non-specific binding - Depends on enrichment efficiency - Complex workflow
Simultaneous epigenetic and genetic sequencing				
SMRT [112,113] (Single molecule, real-time sequencing)	2010	- DNA polymerase incorporates fluorescently labeled nucleotides for real-time sequencing - Detection sensitivity improved if combined with another assay (i.e., HMe-SEAL) - Final readout: determined by the kinetic signature of the base	- Single-base resolution sequencing - Can sequence long reads - Can simultaneously sequence cytosine modifications and genomic sequences - Strand-specific 5hmC sequencing	- Costly - Requires large amounts of input DNA - High sequencing depth
6-letter seq [114]	2023	- Synthetic DNA hairpin adapters bind DNA (paired), split strands, then allow for the synthesis of a complementary strand - One strand from each pair of hairpins will be treated with (1) DNMT5 or (2) T4-βGT glucosylation, oxidation, and deamination - Two-base codes comparing pairwise alignments are used to deconvolute the final readout	- Can simultaneously sequence 5mC, 5hmC, and genomic sequences - Low DNA input requirements - Can use cfDNA as input	- Specific to CpG regions only - High sequencing depth (paired end)
DARESOME [115] (DNA analysis by restriction enzyme for simultaneous detection of multiple epigenomic states)	2023	- Digestion by restriction enzymes (Hpa II, Msp I), followed by ligation with adaptor tags (U-tag, H-tag, M-tag), and glucosylation - Final readout: based on the adaptor tag	- Single-tube workflow - Can be applied to cfDNA or single cells	- Captures CCGG sites only - Restriction enzyme digestion restricts analysis to only one site per DNA fragment
SIMPLE-seq [116] (Single-cell intracellular metabolite profiling and labeling experiment sequencing)	2024	- Relies on a series of “C-to-T” conversions - First “C-to-T” (5hmC): oxidation by ruthenate (VI), labelling by malononitrile, and primer extension to record conversion - Second “C-to-T” (5mC): oxidation by TET, borane reduction - * Final readout (step 1): C, T, T, C, C - * Final readout (step 2): C, C, T, C, C	- Can simultaneously sequence 5mC and 5hmC in a single-cell context - Primer tagging enables multiple readouts using the same sample mixture	- May overestimate 5hmC signal if endogenous 5fC is labelled
Joint-snhmC-seq [117]	2024	- Lysed cells are first treated with sodium bisulfite to fragment DNA (ssDNA) and convert “C-to-T” - To detect 5hmC: ssDNA is deaminated by APOBEC3A (snhmC-seq2) - * Final readout: T, T, C, T, T - To detect 5mC + 5hmC: random priming of ssDNA, followed by adaptor ligation, PCR amplification and sequencing (snmC-seq2) - Similar assay: ACE-seq [103,104]	- Can simultaneously sequence 5mC and 5hmC in a single-cell context - Low DNA input requirements	- Relies on enzyme efficiency - Indirect detection for 5mC - Splits genomic material into two parts for two workflows
Dyad-seq [118]	2024	- Four variants of Dyad-seq designed to profile different combinations of C modifications at CpG dinucleotides - Four steps: ○ (1) T4-βGT glucosylation ○ (2) Enzymatic digestion by MspJI (for 5mC) or AbaSI (for 5hmC) to detect modified C on top strand ○ (3) Adaptor ligation ○ (4) Enzymatic processing ** to detect C modification on bottom strand - * Final readout: T, C, C, T, T	- Can simultaneously sequence 5mC and 5hmC in a single-cell context - Can be further integrated with transcriptomics data	- Resolution limited to CpG - Requires large amounts of DNA input

* Final readout format is as follows: C (cytosine), 5mC (5-methylcytosine), 5hmC (5-hydoxymethylcytosine), 5fC (5-formylcytosine), and 5caC (5-carboxylcytosine). ** Enzymatic processing will depend on the modification being detected. For 5mC, it will undergo oxidation by TET, glucosylation by T4-βGT, deamination by APOBEC3A/sodium bisulfite. For 5hmC, it will undergo glucosylation by T4-βGT, then deamination by APOBEC3A. KRuO_4_ or K_2_RuO_4_ potassium perruthenate; TET, ten-eleven translocation dioxygenase; T4-βGT, T4-β glucosyltransferase; NaIO_4_, sodium metaperiodate; CMS, cytosine-5-methylenesulfonate; APOBEC3A, apolipoprotein B mRNA-editing catalytic polypeptide-like 3A protein; eA3A-v10, engineered human APOBEC3A; DHU, dihydrouridine; DNMT5, NDA methyltransferase 5.

**Table 2:** Examples of DNA Hydroxymethylation as a Cancer Biomarker.

Study	Cancer (n)	Profiling Method	Sample Type	Key Findings
Diagnostic biomarker				
Shao et al., 2022 [78]	Pan cancer (Bladder [n = 41], breast [n = 62], colorectal [n = 45], kidney [n = 54], lung [n = 57], prostate [n = 125))	Nano-hmC-Seal	cfDNA	- Both the pan-cancer weighted diagnostic (wd) model (24 5hmC-gene signature) and the cancer-specific wd model could distinguish between cancer and non-cancer controls - High performance for detecting early-stage disease
Chang et al., 2024 [137]	Colorectal cancer (n = 2576)	HMe-SEAL	cfDNA	- Wd model (96 5hmC-gene body signature) could distinguish between colorectal cancer and non-cancer controls - Wd model outperformed carcinoembryonic antigen (CEA) in detecting colorectal cancer, especially in early-stage disease
Prognostic biomarker				
Dong et al., 2015 [72]	Intrahepatic cholangiocarcinoma (n = 16)	IHC, dot blot, tissue microarray	Tissue	- Low 5hmC levels correlated with higher tumour stage, more lymph node metastases, shorter OS, worse DFS
Fu et al., 2022 [138]	Gastric cancer (n = 144)	ELISA	Tissue	- High 5hmC is an independent predictor of survival, associated with improved OS and lower tumour stage
Kuang et al., 2024 [139]	Endometrial cancer (n = 264)	IHC	Tissue	- *TET2*^−^/5hmC^−^ tumours were associated with the poorest OS - Multivariate analysis revealed that TET2/5hmC relationship can serve as an independent prognostic factor
Chiu et al., 2019 [63]	Diffuse large B-cell lymphoma (n = 48)	HMe-SEAL	cfDNA	- Weighted prognostic (wp) model (29 5hmC-gene signature) had high performance in predicting EFS and OS - High wp-score significantly associated with worse OS and EFS - Wp model outperformed existing prognostic factors (i.e., lactate dehydrogenase, high international prognostic index)
Cai et al., 2021 [64]	Hepatocellular carcinoma (HCC, n = 135)	HMe-SEAL	cfDNA	- HCC score model combining cfDNA 5hmC signatures and HCC protein biomarkers (AFP and DCP) could distinguish between HCC and non-cancer controls - HCC score positively correlated with microvascular invasion, tumour size, tumour stage, greater recurrence rates, shorter RFS, shorter OS, and real-time tumour burden dynamics
Shao et al., 2023 [65]	Acute myeloid leukemia (AML, n = 54)	HMe-SEAL	cfDNA	- Unsupervised hierarchical clustering on the top 500 cell-free 5hmC regions identified three clusters with different risks of AML - Cluster 3 (lowest 5hmC levels on the *TET2* promoter) was associated with the worst OS and highest leukemia burden
Shao et al., 2024 [140]	Lung cancer (n = 97)	Nano-hmC-Seal	cfDNA	- Wp model (17 5hmC-gene signature) demonstrated that a lower wp score was associated with better median OS and PFS - Wp model outperformed well-established clinical factors (i.e., age, sex, smoking history, tumour stage) in predicting survival outcomes
Predictive biomarker				
Guo et al., 2023 [141]	Hepatocellular carcinoma (n = 101)	IHC, tissue microarray	Tissue	- Chemoresistant tissue and cell lines had significantly lower global 5hmC levels and *TET2* expression - Exogenous *TET2* overexpression restored global 5hmC levels and resensitized cells to chemotherapy - Knockdown of *TET2* in chemosensitive cell lines reduced global 5hmC levels - 5hmC loss induces chemoresistance via the PCAF/AKT axis
Chen et al., 2021 [142]	Diffuse large B-cell lymphoma (DLBCL, n = 86)	HMe-SEAL	cfDNA	- Responders and non-responders to chemotherapy had different pre-treatment 5hmC profiles - Logistic model (13 cell-free 5hmC region signature) could accurately identify responders from non-responders
Shao et al., 2024 [66]	Lung cancer (n = 83)	Nano-hmC-Seal	cfDNA	- Wp model (16 5hmC-gene signature) could predict ICI outcomes, in which a lower wp-score was associated with significantly longer median OS and ORR - Wp score outperformed PD-L1 TPS in predicting ICI outcomes
Guler et al., 2024 [143]	Lung cancer (n = 31 with plasma, n = 18 with tissue)	Chemical capture with biotin and streptavidin beads	cfDNA	- In ICI responders, 5hmC levels were elevated at immune cell activating genes and reduced at drug resistance genes - In ICI non-responders, 5hmC levels were elevated in ICI resistance genes and reduced in immune genes
Multi-omics biomarker				
Hu et al., 2022 [126]	Lung cancer (n = 157)	HMe-SEAL	cfDNA	- Integrated fragment length and 5hmC model for early lung cancer detection (37 5hmC markers, 48 fragmentation features) - Integrated model outperformed standalone 5hmC and fragmentomics models
Zhang et al., 2023 [144]	Pan cancer (Liver [n = 132], pancreas [n = 74], lung [n = 33], glioblastoma [n = 33])	HMe-SEAL	cfDNA	- Integrated cfDNA fragment features (i.e., size, coverage, preferred ends) and 65 5hmC signatures for detecting cancer - Integrated model had high performance, sensitivity and specificity
Shi et al., 2023 [145]	Bladder cancer (n = 44)	RRBS, oxRRBS	cfDNA	- Alterations in 5hmC and 5mC were associated with bladder cancer recurrence and tumour immune escape pathways
Lee et al., 2024 [47]	Pediatric central nervous system tumours (n = 32)	Infinium Human-Methylation EPIC BeadChips OxBS-seq	cfDNA	- Tumour composition and heterogeneity influences the abundance and alterations in 5mC and 5hmC

5hmC, hydroxymethylation; 5mC, methylation; OS, overall survival; DFS, disease-free survival; EFS, event-free survival; PFS, progress-free survival; RFS, relapse-free survival; ORR, objective response rate; DCB, durable clinical benefit; AFP, alpha-fetoprotein; DCP, des-gamma-carboxy prothrombin; ICI, immune checkpoint blockade; PD-L1, programmed death-ligand 1; TPS, tumour proportion score.

## References

[1] Thomson J.P., Meehan R.R. (2017). The Application of Genome-Wide 5-Hydroxymethylcytosine Studies in Cancer Research. Epigenomics.

[2] Pfeifer G.P., Kadam S., Jin S.-G. (2013). 5-Hydroxymethylcytosine and Its Potential Roles in Development and Cancer. Epigenet. Chromatin.

[3] Kudo Y., Tateishi K., Yamamoto K., Yamamoto S., Asaoka Y., Ijichi H., Nagae G., Yoshida H., Aburatani H., Koike K. (2012). Loss of 5-hydroxymethylcytosine Is Accompanied with Malignant Cellular Transformation. Cancer Sci..

[4] García-Pardo M., Makarem M., Li J.J.N., Kelly D., Leighl N.B. (2022). Integrating Circulating-Free DNA (cfDNA) Analysis into Clinical Practice: Opportunities and Challenges. Br. J. Cancer.

[5] Cescon D.W., Bratman S.V., Chan S.M., Siu L.L. (2020). Circulating Tumor DNA and Liquid Biopsy in Oncology. Nat. Cancer.

[6] Xu T., Gao H. (2020). Hydroxymethylation and Tumors: Can 5-Hydroxymethylation Be Used as a Marker for Tumor Diagnosis and Treatment?. Hum. Genom..

[7] Xu L., Zhou Y., Chen L., Bissessur A.S., Chen J., Mao M., Ju S., Chen L., Chen C., Li Z. (2021). Deoxyribonucleic Acid 5-Hydroxymethylation in Cell-Free Deoxyribonucleic Acid, a Novel Cancer Biomarker in the Era of Precision Medicine. Front. Cell Dev. Biol..

[8] Besaratinia A., Caceres A., Tommasi S. (2022). DNA Hydroxymethylation in Smoking-Associated Cancers. Int. J. Mol. Sci..

[9] Smith Z.D., Meissner A. (2013). DNA Methylation: Roles in Mammalian Development. Nat. Rev. Genet..

[10] Ooi S.K.T., O’Donnell A.H., Bestor T.H. (2009). Mammalian Cytosine Methylation at a Glance. J. Cell Sci..

[11] Tahiliani M., Koh K.P., Shen Y., Pastor W.A., Bandukwala H., Brudno Y., Agarwal S., Iyer L.M., Liu D.R., Aravind L. (2009). Conversion of 5-Methylcytosine to 5-Hydroxymethylcytosine in Mammalian DNA by MLL Partner TET1. Science.

[12] Ito S., Shen L., Dai Q., Wu S.C., Collins L.B., Swenberg J.A., He C., Zhang Y. (2011). Tet Proteins Can Convert 5-Methylcytosine to 5-Formylcytosine and 5-Carboxylcytosine. Science.

[13] Wu X., Zhang Y. (2017). TET-Mediated Active DNA Demethylation: Mechanism, Function and Beyond. Nat. Rev. Genet..

[14] Globisch D., Münzel M., Müller M., Michalakis S., Wagner M., Koch S., Brückl T., Biel M., Carell T. (2010). Tissue Distribution of 5-Hydroxymethylcytosine and Search for Active Demethylation Intermediates. PLoS ONE.

[15] Hu L., Lu J., Cheng J., Rao Q., Li Z., Hou H., Lou Z., Zhang L., Li W., Gong W. (2015). Structural Insight into Substrate Preference for TET-Mediated Oxidation. Nature.

[16] Hashimoto H., Hong S., Bhagwat A.S., Zhang X., Cheng X. (2012). Excision of 5-Hydroxymethyluracil and 5-Carboxylcytosine by the Thymine DNA Glycosylase Domain: Its Structural Basis and Implications for Active DNA Demethylation. Nucleic Acids Res..

[17] Nabel C.S., Jia H., Ye Y., Shen L., Goldschmidt H.L., Stivers J.T., Zhang Y., Kohli R.M. (2012). AID/APOBEC Deaminases Disfavor Modified Cytosines Implicated in DNA Demethylation. Nat. Chem. Biol..

[18] Kohli R.M., Zhang Y. (2013). TET Enzymes, TDG and the Dynamics of DNA Demethylation. Nature.

[19] He Y.-F., Li B.-Z., Li Z., Liu P., Wang Y., Tang Q., Ding J., Jia Y., Chen Z., Li L. (2011). Tet-Mediated Formation of 5-Carboxylcytosine and Its Excision by TDG in Mammalian DNA. Science.

[20] Maiti A., Drohat A.C. (2011). Thymine DNA Glycosylase Can Rapidly Excise 5-Formylcytosine and 5-Carboxylcytosine: Potential Implications for Active Demethylation of CpG Sites. J. Biol. Chem..

[21] Bachman M., Uribe-Lewis S., Yang X., Williams M., Murrell A., Balasubramanian S. (2014). 5-Hydroxymethylcytosine Is a Predominantly Stable DNA Modification. Nat. Chem..

[22] Ito S., D’Alessio A.C., Taranova O.V., Hong K., Sowers L.C., Zhang Y. (2010). Role of Tet Proteins in 5mC to 5hmC Conversion, ES-Cell Self-Renewal and Inner Cell Mass Specification. Nature.

[23] Ficz G., Branco M.R., Seisenberger S., Santos F., Krueger F., Hore T.A., Marques C.J., Andrews S., Reik W. (2011). Dynamic Regulation of 5-Hydroxymethylcytosine in Mouse ES Cells and during Differentiation. Nature.

[24] Guibert S., Weber M. (2013). Functions of DNA Methylation and Hydroxymethylation in Mammalian Development. Curr. Top. Dev. Biol..

[25] Dawlaty M.M., Breiling A., Le T., Barrasa M.I., Raddatz G., Gao Q., Powell B.E., Cheng A.W., Faull K.F., Lyko F. (2014). Loss of Tet Enzymes Compromises Proper Differentiation of Embryonic Stem Cells. Dev. Cell.

[26] Vasconcelos S., Caniçais C., Chuva de Sousa Lopes S.M., Marques C.J., Dória S. (2023). The Role of DNA Hydroxymethylation and TET Enzymes in Placental Development and Pregnancy Outcome. Clin. Epigenet..

[27] Gao Y., Chen J., Li K., Wu T., Huang B., Liu W., Kou X., Zhang Y., Huang H., Jiang Y. (2013). Replacement of Oct4 by Tet1 during iPSC Induction Reveals an Important Role of DNA Methylation and Hydroxymethylation in Reprogramming. Cell Stem Cell.

[28] Neri F., Incarnato D., Krepelova A., Dettori D., Rapelli S., Maldotti M., Parlato C., Anselmi F., Galvagni F., Oliviero S. (2015). TET1 Is Controlled by Pluripotency-Associated Factors in ESCs and Downmodulated by PRC2 in Differentiated Cells and Tissues. Nucleic Acids Res..

[29] Wu X., Li G., Xie R. (2018). Decoding the Role of TET Family Dioxygenases in Lineage Specification. Epigenet. Chromatin.

[30] Senner C.E., Chrysanthou S., Burge S., Lin H.-Y., Branco M.R., Hemberger M. (2020). TET1 and 5-Hydroxymethylation Preserve the Stem Cell State of Mouse Trophoblast. Stem Cell Rep..

[31] Cheng Y., Xie N., Jin P., Wang T. (2015). DNA Methylation and Hydroxymethylation in Stem Cells. Cell Biochem. Funct..

[32] Tsagaratou A., Äijö T., Lio C.-W.J., Yue X., Huang Y., Jacobsen S.E., Lähdesmäki H., Rao A. (2014). Dissecting the Dynamic Changes of 5-Hydroxymethylcytosine in T-Cell Development and Differentiation. Proc. Natl. Acad. Sci. USA.

[33] Tsiouplis N.J., Bailey D.W., Chiou L.F., Wissink F.J., Tsagaratou A. (2020). TET-Mediated Epigenetic Regulation in Immune Cell Development and Disease. Front. Cell Dev. Biol..

[34] Xiao Q., Nobre A., Piñeiro P., Berciano-Guerrero M.-Á., Alba E., Cobo M., Lauschke V.M., Barragán I. (2020). Genetic and Epigenetic Biomarkers of Immune Checkpoint Blockade Response. J. Clin. Med..

[35] McPherson R.C., Konkel J.E., Prendergast C.T., Thomson J.P., Ottaviano R., Leech M.D., Kay O., Zandee S.E.J., Sweenie C.H., Wraith D.C. (2014). Epigenetic Modification of the PD-1 (Pdcd1) Promoter in Effector CD4(+) T Cells Tolerized by Peptide Immunotherapy. eLife.

[36] Khoodoruth M.A.S., Khoodoruth W.N.C., Alwani R.A. (2024). Exploring the Epigenetic Landscape: The Role of 5-Hydroxymethylcytosine in Neurodevelopmental Disorders. Camb. Prisms Precis. Med..

[37] Cui X.-L., Nie J., Ku J., Dougherty U., West-Szymanski D.C., Collin F., Ellison C.K., Sieh L., Ning Y., Deng Z. (2020). A Human Tissue Map of 5-Hydroxymethylcytosines Exhibits Tissue Specificity through Gene and Enhancer Modulation. Nat. Commun..

[38] Stroud H., Feng S., Morey Kinney S., Pradhan S., Jacobsen S.E. (2011). 5-Hydroxymethylcytosine Is Associated with Enhancers and Gene Bodies in Human Embryonic Stem Cells. Genome Biol..

[39] Szulwach K.E., Li X., Li Y., Song C.-X., Han J.W., Kim S., Namburi S., Hermetz K., Kim J.J., Rudd M.K. (2011). Integrating 5-Hydroxymethylcytosine into the Epigenomic Landscape of Human Embryonic Stem Cells. PLoS Genet..

[40] Pastor W.A., Pape U.J., Huang Y., Henderson H.R., Lister R., Ko M., McLoughlin E.M., Brudno Y., Mahapatra S., Kapranov P. (2011). Genome-Wide Mapping of 5-Hydroxymethylcytosine in Embryonic Stem Cells. Nature.

[41] Kranzhöfer D.K., Gilsbach R., Grüning B.A., Backofen R., Nührenberg T.G., Hein L. (2016). 5′-Hydroxymethylcytosine Precedes Loss of CpG Methylation in Enhancers and Genes Undergoing Activation in Cardiomyocyte Maturation. PLoS ONE.

[42] Uribe-Lewis S., Stark R., Carroll T., Dunning M.J., Bachman M., Ito Y., Stojic L., Halim S., Vowler S.L., Lynch A.G. (2015). 5-Hydroxymethylcytosine Marks Promoters in Colon That Resist DNA Hypermethylation in Cancer. Genome Biol..

[43] Lee J., Lee D., Kim H.-P., Kim T.-Y., Bang D. (2023). EBS-Seq: Enrichment-Based Method for Accurate Analysis of 5-Hydroxymethylcytosine at Single-Base Resolution. Clin. Epigenet..

[44] Han D., Lu X., Shih A.H., Nie J., You Q., Xu M.M., Melnick A.M., Levine R.L., He C. (2016). A Highly Sensitive and Robust Method for Genome-Wide 5hmC Profiling of Rare Cell Populations. Mol. Cell.

[45] Branco M.R., Ficz G., Reik W. (2011). Uncovering the Role of 5-Hydroxymethylcytosine in the Epigenome. Nat. Rev. Genet..

[46] Wu H., D’Alessio A.C., Ito S., Wang Z., Cui K., Zhao K., Sun Y.E., Zhang Y. (2011). Genome-Wide Analysis of 5-Hydroxymethylcytosine Distribution Reveals Its Dual Function in Transcriptional Regulation in Mouse Embryonic Stem Cells. Genes Dev..

[47] Lee M.K., Azizgolshani N., Zhang Z., Perreard L., Kolling F.W., Nguyen L.N., Zanazzi G.J., Salas L.A., Christensen B.C. (2024). Associations in Cell Type-Specific Hydroxymethylation and Transcriptional Alterations of Pediatric Central Nervous System Tumors. Nat. Commun..

[48] Valinluck V., Tsai H.-H., Rogstad D.K., Burdzy A., Bird A., Sowers L.C. (2004). Oxidative Damage to Methyl-CpG Sequences Inhibits the Binding of the Methyl-CpG Binding Domain (MBD) of Methyl-CpG Binding Protein 2 (MeCP2). Nucleic Acids Res..

[49] Mellén M., Ayata P., Dewell S., Kriaucionis S., Heintz N. (2012). MeCP2 Binds to 5hmC Enriched within Active Genes and Accessible Chromatin in the Nervous System. Cell.

[50] Shi D.-Q., Ali I., Tang J., Yang W.-C. (2017). New Insights into 5hmC DNA Modification: Generation, Distribution and Function. Front. Genet..

[51] He B., Zhang C., Zhang X., Fan Y., Zeng H., Liu J., Meng H., Bai D., Peng J., Zhang Q. (2021). Tissue-Specific 5-Hydroxymethylcytosine Landscape of the Human Genome. Nat. Commun..

[52] Nestor C.E., Ottaviano R., Reddington J., Sproul D., Reinhardt D., Dunican D., Katz E., Dixon J.M., Harrison D.J., Meehan R.R. (2012). Tissue Type Is a Major Modifier of the 5-Hydroxymethylcytosine Content of Human Genes. Genome Res..

[53] Li W., Liu M. (2011). Distribution of 5-Hydroxymethylcytosine in Different Human Tissues. J. Nucleic Acids.

[54] Song C.-X., Szulwach K.E., Fu Y., Dai Q., Yi C., Li X., Li Y., Chen C.-H., Zhang W., Jian X. (2011). Selective Chemical Labeling Reveals the Genome-Wide Distribution of 5-Hydroxymethylcytosine. Nat. Biotechnol..

[55] Haffner M.C., Chaux A., Meeker A.K., Esopi D.M., Gerber J., Pellakuru L.G., Toubaji A., Argani P., Iacobuzio-Donahue C., Nelson W.G. (2011). Global 5-Hydroxymethylcytosine Content Is Significantly Reduced in Tissue Stem/Progenitor Cell Compartments and in Human Cancers. Oncotarget.

[56] Munari E., Chaux A., Vaghasia A.M., Taheri D., Karram S., Bezerra S.M., Gonzalez Roibon N., Nelson W.G., Yegnasubramanian S., Netto G.J. (2016). Global 5-Hydroxymethylcytosine Levels Are Profoundly Reduced in Multiple Genitourinary Malignancies. PLoS ONE.

[57] Gambichler T., Sand M., Skrygan M. (2013). Loss of 5-Hydroxymethylcytosine and Ten-Eleven Translocation 2 Protein Expression in Malignant Melanoma. Melanoma Res..

[58] Song C.-X., Yin S., Ma L., Wheeler A., Chen Y., Zhang Y., Liu B., Xiong J., Zhang W., Hu J. (2017). 5-Hydroxymethylcytosine Signatures in Cell-Free DNA Provide Information about Tumor Types and Stages. Cell Res..

[59] Li W., Zhang X., Lu X., You L., Song Y., Luo Z., Zhang J., Nie J., Zheng W., Xu D. (2017). 5-Hydroxymethylcytosine Signatures in Circulating Cell-Free DNA as Diagnostic Biomarkers for Human Cancers. Cell Res..

[60] Zhang J., Han X., Gao C., Xing Y., Qi Z., Liu R., Wang Y., Zhang X., Yang Y.-G., Li X. (2018). 5-Hydroxymethylome in Circulating Cell-Free DNA as A Potential Biomarker for Non-Small-Cell Lung Cancer. Genom. Proteom. Bioinform..

[61] Cai J., Chen L., Zhang Z., Zhang X., Lu X., Liu W., Shi G., Ge Y., Gao P., Yang Y. (2019). Genome-Wide Mapping of 5-Hydroxymethylcytosines in Circulating Cell-Free DNA as a Non-Invasive Approach for Early Detection of Hepatocellular Carcinoma. Gut.

[62] Guler G.D., Ning Y., Ku C.-J., Phillips T., McCarthy E., Ellison C.K., Bergamaschi A., Collin F., Lloyd P., Scott A. (2020). Detection of Early Stage Pancreatic Cancer Using 5-Hydroxymethylcytosine Signatures in Circulating Cell Free DNA. Nat. Commun..

[63] Chiu B.C.-H., Zhang Z., You Q., Zeng C., Stepniak E., Bracci P.M., Yu K., Venkataraman G., Smith S.M., He C. (2019). Prognostic Implications of 5-Hydroxymethylcytosines from Circulating Cell-Free DNA in Diffuse Large B-Cell Lymphoma. Blood Adv..

[64] Cai Z., Zhang J., He Y., Xia L., Dong X., Chen G., Zhou Y., Hu X., Zhong S., Wang Y. (2021). Liquid Biopsy by Combining 5-Hydroxymethylcytosine Signatures of Plasma Cell-Free DNA and Protein Biomarkers for Diagnosis and Prognosis of Hepatocellular Carcinoma. ESMO Open.

[65] Shao J., Shah S., Ganguly S., Zu Y., He C., Li Z. (2023). Classification of Acute Myeloid Leukemia by Cell-Free DNA 5-Hydroxymethylcytosine. Genes.

[66] Shao J., Xu Y., Olsen R.J., Kasparian S., Sun K., Mathur S., Zhang J., He C., Chen S.-H., Bernicker E.H. (2024). 5-Hydroxymethylcytosine in Cell-Free DNA Predicts Immunotherapy Response in Lung Cancer. Cells.

[67] Gao P., Lin S., Cai M., Zhu Y., Song Y., Sui Y., Lin J., Liu J., Lu X., Zhong Y. (2019). 5-Hydroxymethylcytosine Profiling from Genomic and Cell-Free DNA for Colorectal Cancers Patients. J. Cell. Mol. Med..

[68] Peng D., Ge G., Gong Y., Zhan Y., He S., Guan B., Li Y., Xu Z., Hao H., He Z. (2018). Vitamin C Increases 5-Hydroxymethylcytosine Level and Inhibits the Growth of Bladder Cancer. Clin. Epigenet..

[69] Applebaum M.A., Barr E.K., Karpus J., Nie J., Zhang Z., Armstrong A.E., Uppal S., Sukhanova M., Zhang W., Chlenski A. (2019). 5-Hydroxymethylcytosine Profiles Are Prognostic of Outcome in Neuroblastoma and Reveal Transcriptional Networks That Correlate with Tumor Phenotype. JCO Precis. Oncol..

[70] Ramasamy D., Rao A.K.D.M., Balaiah M., Vittal Rangan A., Sundersingh S., Veluswami S., Thangarajan R., Mani S. (2022). Locus-Specific Enrichment Analysis of 5-Hydroxymethylcytosine Reveals Novel Genes Associated with Breast Carcinogenesis. Cells.

[71] Chen Z., Shi X., Guo L., Li Y., Luo M., He J. (2016). Decreased 5-Hydroxymethylcytosine Levels Correlate with Cancer Progression and Poor Survival: A Systematic Review and Meta-Analysis. Oncotarget.

[72] Dong Z.-R., Zhang C., Cai J.-B., Zhang P.-F., Shi G.-M., Gao D.-M., Sun H.-C., Qiu S.-J., Zhou J., Ke A.-W. (2015). Role of 5-Hydroxymethylcytosine Level in Diagnosis and Prognosis Prediction of Intrahepatic Cholangiocarcinoma. Tumour Biol. J. Int. Soc. Oncodev. Biol. Med..

[73] Liao Y., Gu J., Wu Y., Long X., Ge D.I., Xu J., Ding J. (2016). Low Level of 5-Hydroxymethylcytosine Predicts Poor Prognosis in Non-Small Cell Lung Cancer. Oncol. Lett..

[74] Zhang Y., Wu K., Shao Y., Sui F., Yang Q., Shi B., Hou P., Ji M. (2016). Decreased 5-Hydroxymethylcytosine (5-hmC) Predicts Poor Prognosis in Early-Stage Laryngeal Squamous Cell Carcinoma. Am. J. Cancer Res..

[75] Qiu L., Liu F., Yi S., Li X., Liu X., Xiao C., Lian C.G., Tu P., Wang Y. (2018). Loss of 5-Hydroxymethylcytosine Is an Epigenetic Biomarker in Cutaneous T-Cell Lymphoma. J. Investig. Dermatol..

[76] Ren Y., Zhang Z., She Y., He Y., Li D., Shi Y., He C., Yang Y., Zhang W., Chen C. (2023). A Highly Sensitive and Specific Non-Invasive Test through Genome-Wide 5-Hydroxymethylation Mapping for Early Detection of Lung Cancer. Small Methods.

[77] Bhattacharyya S., Pradhan K., Campbell N., Mazdo J., Vasantkumar A., Maqbool S., Bhagat T.D., Gupta S., Suzuki M., Yu Y. (2017). Altered Hydroxymethylation Is Seen at Regulatory Regions in Pancreatic Cancer and Regulates Oncogenic Pathways. Genome Res..

[78] Shao J., Bernicker E.H., He C., Li Z. (2022). Cell-Free DNA 5-Hydroxymethylcytosine as a Marker for Common Cancer Detection. Clin. Transl. Discov..

[79] Sjöström M., Zhao S.G., Levy S., Zhang M., Ning Y., Shrestha R., Lundberg A., Herberts C., Foye A., Aggarwal R. (2022). The 5-Hydroxymethylcytosine Landscape of Prostate Cancer. Cancer Res..

[80] Fu Y., Jiang J., Wu Y., Cao D., Jia Z., Zhang Y., Li D., Cui Y., Zhang Y., Cao X. (2024). Genome-Wide 5-Hydroxymethylcytosines in Circulating Cell-Free DNA as Noninvasive Diagnostic Markers for Gastric Cancer. Gastric Cancer Off. J. Int. Gastric Cancer Assoc. Jpn. Gastric Cancer Assoc..

[81] Wagner M., Steinbacher J., Kraus T.F.J., Michalakis S., Hackner B., Pfaffeneder T., Perera A., Müller M., Giese A., Kretzschmar H.A. (2015). Age-Dependent Levels of 5-Methyl-, 5-Hydroxymethyl-, and 5-Formylcytosine in Human and Mouse Brain Tissues. Angew. Chem. Int. Ed..

[82] Kriaucionis S., Heintz N. (2009). The Nuclear DNA Base 5-Hydroxymethylcytosine Is Present in Purkinje Neurons and the Brain. Science.

[83] Kroeze L.I., Aslanyan M.G., van Rooij A., Koorenhof-Scheele T.N., Massop M., Carell T., Boezeman J.B., Marie J.-P., Halkes C.J.M., de Witte T. (2014). Characterization of Acute Myeloid Leukemia Based on Levels of Global Hydroxymethylation. Blood.

[84] Shahal T., Green O., Hananel U., Michaeli Y., Shabat D., Ebenstein Y. (2016). Simple and Cost-Effective Fluorescent Labeling of 5-Hydroxymethylcytosine. Methods Appl. Fluoresc..

[85] Berney M., McGouran J.F. (2018). Methods for Detection of Cytosine and Thymine Modifications in DNA. Nat. Rev. Chem..

[86] Zeng C., Stroup E.K., Zhang Z., Chiu B.C.-H., Zhang W. (2019). Towards Precision Medicine: Advances in 5-Hydroxymethylcytosine Cancer Biomarker Discovery in Liquid Biopsy. Cancer Commun..

[87] Wang T., Loo C.E., Kohli R.M. (2022). Enzymatic Approaches for Profiling Cytosine Methylation and Hydroxymethylation. Mol. Metab..

[88] He B., Yao H., Yi C. (2024). Advances in the Joint Profiling Technologies of 5mC and 5hmC. RSC Chem. Biol..

[89] Frommer M., McDonald L.E., Millar D.S., Collis C.M., Watt F., Grigg G.W., Molloy P.L., Paul C.L. (1992). A Genomic Sequencing Protocol That Yields a Positive Display of 5-Methylcytosine Residues in Individual DNA Strands. Proc. Natl. Acad. Sci. USA.

[90] Booth M.J., Branco M.R., Ficz G., Oxley D., Krueger F., Reik W., Balasubramanian S. (2012). Quantitative Sequencing of 5-Methylcytosine and 5-Hydroxymethylcytosine at Single-Base Resolution. Science.

[91] Stewart S.K., Morris T.J., Guilhamon P., Bulstrode H., Bachman M., Balasubramanian S., Beck S. (2015). oxBS-450K: A Method for Analysing Hydroxymethylation Using 450K BeadChips. Methods.

[92] Matsushita T., Moriyama Y., Nagae G., Aburatani H., Okamoto A. (2017). DNA-Friendly Cu(Ii)/TEMPO-Catalyzed 5-Hydroxymethylcytosine-Specific Oxidation. Chem. Commun..

[93] Fukuzawa S., Takahashi S., Tachibana K., Tajima S., Suetake I. (2016). Simple and Accurate Single Base Resolution Analysis of 5-Hydroxymethylcytosine by Catalytic Oxidative Bisulfite Sequencing Using Micelle Incarcerated Oxidants. Bioorg. Med. Chem..

[94] Skvortsova K., Zotenko E., Luu P.-L., Gould C.M., Nair S.S., Clark S.J., Stirzaker C. (2017). Comprehensive Evaluation of Genome-Wide 5-Hydroxymethylcytosine Profiling Approaches in Human DNA. Epigenet. Chromatin.

[95] Yu M., Hon G.C., Szulwach K.E., Song C.-X., Zhang L., Kim A., Li X., Dai Q., Shen Y., Park B. (2012). Base-Resolution Analysis of 5-Hydroxymethylcytosine in the Mammalian Genome. Cell.

[96] Moran S., Arribas C., Esteller M. (2016). Validation of a DNA Methylation Microarray for 850,000 CpG Sites of the Human Genome Enriched in Enhancer Sequences. Epigenomics.

[97] Hahn M.A., Li A.X., Wu X., Pfeifer G.P. (2015). Single Base Resolution Analysis of 5-Methylcytosine and 5-Hydroxymethylcytosine by RRBS and TAB-RRBS. Methods Mol. Biol..

[98] Pastor W.A., Huang Y., Henderson H.R., Agarwal S., Rao A. (2012). The GLIB Technique for Genome-Wide Mapping of 5-Hydroxymethylcytosine. Nat. Protoc..

[99] Robertson A.B., Dahl J.A., Ougland R., Klungland A. (2012). Pull-down of 5-Hydroxymethylcytosine DNA Using JBP1-Coated Magnetic Beads. Nat. Protoc..

[100] Cui L., Chung T.H., Tan D., Sun X., Jia X.-Y. (2014). JBP1-Seq: A Fast and Efficient Method for Genome-Wide Profiling of 5hmC. Genomics.

[101] Nestor C.E., Meehan R.R. (2014). Hydroxymethylated DNA Immunoprecipitation (hmeDIP). Methods Mol. Biol..

[102] Huang Y., Pastor W.A., Zepeda-Martínez J.A., Rao A. (2012). The Anti-CMS Technique for Genome-Wide Mapping of 5-Hydroxymethylcytosine. Nat. Protoc..

[103] Schutsky E.K., DeNizio J.E., Hu P., Liu M.Y., Nabel C.S., Fabyanic E.B., Hwang Y., Bushman F.D., Wu H., Kohli R.M. (2018). Nondestructive, Base-Resolution Sequencing of 5-Hydroxymethylcytosine Using a DNA Deaminase. Nat. Biotechnol..

[104] Skvortsova K., Bogdanovic O. (2021). TAB-Seq and ACE-Seq Data Processing for Genome-Wide DNA Hydroxymethylation Profiling. Methods Mol. Biol..

[105] Vaisvila R., Ponnaluri V.K.C., Sun Z., Langhorst B.W., Saleh L., Guan S., Dai N., Campbell M.A., Sexton B.S., Marks K. (2021). Enzymatic Methyl Sequencing Detects DNA Methylation at Single-Base Resolution from Picograms of DNA. Genome Res..

[106] Zeng H., He B., Xia B., Bai D., Lu X., Cai J., Chen L., Zhou A., Zhu C., Meng H. (2018). Bisulfite-Free, Nanoscale Analysis of 5-Hydroxymethylcytosine at Single Base Resolution. J. Am. Chem. Soc..

[107] Liu Y., Siejka-Zielińska P., Velikova G., Bi Y., Yuan F., Tomkova M., Bai C., Chen L., Schuster-Böckler B., Song C.-X. (2019). Bisulfite-Free Direct Detection of 5-Methylcytosine and 5-Hydroxymethylcytosine at Base Resolution. Nat. Biotechnol..

[108] Hu L., Liu Y., Han S., Yang L., Cui X., Gao Y., Dai Q., Lu X., Kou X., Zhao Y. (2019). Jump-Seq: Genome-Wide Capture and Amplification of 5-Hydroxymethylcytosine Sites. J. Am. Chem. Soc..

[109] Gibas P., Narmontė M., Staševskij Z., Gordevičius J., Klimašauskas S., Kriukienė E. (2020). Precise Genomic Mapping of 5-Hydroxymethylcytosine via Covalent Tether-Directed Sequencing. PLoS Biol..

[110] Li X., Shi X., Gong Y., Guo W., Liu Y., Peng C., Xu Y. (2021). Selective Chemical Labeling and Sequencing of 5-Hydroxymethylcytosine in DNA at Single-Base Resolution. Front. Genet..

[111] Xie N.-B., Wang M., Chen W., Ji T.-T., Guo X., Gang F.-Y., Wang Y.-F., Feng Y.-Q., Liang Y., Ci W. (2023). Whole-Genome Sequencing of 5-Hydroxymethylcytosine at Base Resolution by Bisulfite-Free Single-Step Deamination with Engineered Cytosine Deaminase. ACS Cent. Sci..

[112] Flusberg B.A., Webster D.R., Lee J.H., Travers K.J., Olivares E.C., Clark T.A., Korlach J., Turner S.W. (2010). Direct Detection of DNA Methylation during Single-Molecule, Real-Time Sequencing. Nat. Methods.

[113] Song C.-X., Clark T.A., Lu X.-Y., Kislyuk A., Dai Q., Turner S.W., He C., Korlach J. (2011). Sensitive and Specific Single-Molecule Sequencing of 5-Hydroxymethylcytosine. Nat. Methods.

[114] Füllgrabe J., Gosal W.S., Creed P., Liu S., Lumby C.K., Morley D.J., Ost T.W.B., Vilella A.J., Yu S., Bignell H. (2023). Simultaneous Sequencing of Genetic and Epigenetic Bases in DNA. Nat. Biotechnol..

[115] Viswanathan R., Cheruba E., Wong P.-M., Yi Y., Ngang S., Chong D.Q., Loh Y.-H., Tan I.B., Cheow L.F. (2023). DARESOME Enables Concurrent Profiling of Multiple DNA Modifications with Restriction Enzymes in Single Cells and Cell-Free DNA. Sci. Adv..

[116] Bai D., Zhang X., Xiang H., Guo Z., Zhu C., Yi C. (2024). Simultaneous Single-Cell Analysis of 5mC and 5hmC with SIMPLE-Seq. Nat. Biotechnol..

[117] Fabyanic E.B., Hu P., Qiu Q., Berríos K.N., Connolly D.R., Wang T., Flournoy J., Zhou Z., Kohli R.M., Wu H. (2024). Joint Single-Cell Profiling Resolves 5mC and 5hmC and Reveals Their Distinct Gene Regulatory Effects. Nat. Biotechnol..

[118] Chialastri A., Sarkar S., Schauer E.E., Lamba S., Dey S.S. (2024). Combinatorial Quantification of 5mC and 5hmC at Individual CpG Dyads and the Transcriptome in Single Cells Reveals Modulators of DNA Methylation Maintenance Fidelity. Nat. Struct. Mol. Biol..

[119] Huang Y., Pastor W.A., Shen Y., Tahiliani M., Liu D.R., Rao A. (2010). The Behaviour of 5-Hydroxymethylcytosine in Bisulfite Sequencing. PLoS ONE.

[120] Li X., Liu Y., Salz T., Hansen K.D., Feinberg A. (2016). Whole-Genome Analysis of the Methylome and Hydroxymethylome in Normal and Malignant Lung and Liver. Genome Res..

[121] Booth M.J., Ost T.W.B., Beraldi D., Bell N.M., Branco M.R., Reik W., Balasubramanian S. (2013). Oxidative Bisulfite Sequencing of 5-Methylcytosine and 5-Hydroxymethylcytosine. Nat. Protoc..

[122] Janku F., Huang H.J., Pereira D.Y., Kobayashi M., Chiu C.H., Call S.G., Woodbury K.T., Chao F., Marshak D.R., Chiu R.Y.T. (2021). A Novel Method for Liquid-Phase Extraction of Cell-Free DNA for Detection of Circulating Tumor DNA. Sci. Rep..

[123] Tanaka K., Okamoto A. (2007). Degradation of DNA by Bisulfite Treatment. Bioorg. Med. Chem. Lett..

[124] Szwagierczak A., Bultmann S., Schmidt C.S., Spada F., Leonhardt H. (2010). Sensitive Enzymatic Quantification of 5-Hydroxymethylcytosine in Genomic DNA. Nucleic Acids Res..

[125] Shahal T., Koren O., Shefer G., Stern N., Ebenstein Y. (2018). Hypersensitive Quantification of Global 5-Hydroxymethylcytosine by Chemoenzymatic Tagging. Anal. Chim. Acta.

[126] Hu X., Luo K., Shi H., Yan X., Huang R., Zhao B., Zhang J., Xie D., Zhang W. (2022). Integrated 5-Hydroxymethylcytosine and Fragmentation Signatures as Enhanced Biomarkers in Lung Cancer. Clin. Epigenet..

[127] Thomson J.P., Hunter J.M., Nestor C.E., Dunican D.S., Terranova R., Moggs J.G., Meehan R.R. (2013). Comparative Analysis of Affinity-Based 5-Hydroxymethylation Enrichment Techniques. Nucleic Acids Res..

[128] Gordevičius J., Narmontė M., Gibas P., Kvederavičiūtė K., Tomkutė V., Paluoja P., Krjutškov K., Salumets A., Kriukienė E. (2020). Identification of Fetal Unmodified and 5-Hydroxymethylated CG Sites in Maternal Cell-Free DNA for Non-Invasive Prenatal Testing. Clin. Epigenet..

[129] Siriwardena S., Chen K., Bhagwat A.S. (2016). The Functions and Malfunctions of AID/APOBEC Family Deaminases: The Known Knowns and the Known Unknowns. Chem. Rev..

[130] Siejka-Zielińska P., Cheng J., Jackson F., Liu Y., Soonawalla Z., Reddy S., Silva M., Puta L., McCain M.V., Culver E.L. (2021). Cell-Free DNA TAPS Provides Multimodal Information for Early Cancer Detection. Sci. Adv..

[131] Chen H.-Q., Chen D.-J., Li Y., Yuan W.-B., Fan J., Zhang Z., Han F., Jiang X., Chen J.-P., Wang D.-D. (2021). Epigenetic Silencing of TET1 Mediated Hydroxymethylation of Base Excision Repair Pathway during Lung Carcinogenesis. Environ. Pollut..

[132] Gabrieli T., Sharim H., Nifker G., Jeffet J., Shahal T., Arielly R., Levi-Sakin M., Hoch L., Arbib N., Michaeli Y. (2018). Epigenetic Optical Mapping of 5-Hydroxymethylcytosine in Nanochannel Arrays. ACS Nano.

[133] Laszlo A.H., Derrington I.M., Brinkerhoff H., Langford K.W., Nova I.C., Samson J.M., Bartlett J.J., Pavlenok M., Gundlach J.H. (2013). Detection and Mapping of 5-Methylcytosine and 5-Hydroxymethylcytosine with Nanopore MspA. Proc. Natl. Acad. Sci. USA.

[134] Du Y., Wang Y., Hu X., Liu J., Diao J. (2020). Single-Molecule Quantification of 5-Methylcytosine and 5-Hydroxymethylcytosine in Cancer Genome. View.

[135] Scarano C., Veneruso I., De Simone R.R., Di Bonito G., Secondino A., D’Argenio V. (2024). The Third-Generation Sequencing Challenge: Novel Insights for the Omic Sciences. Biomolecules.

[136] Cao Y., Bai Y., Yuan T., Song L., Fan Y., Ren L., Song W., Peng J., An R., Gu Q. (2023). Single-Cell Bisulfite-Free 5mC and 5hmC Sequencing with High Sensitivity and Scalability. Proc. Natl. Acad. Sci. USA.

[137] Chang W., Zhang Z., Jia B., Ding K.-F., Pan Z., Su G., Zhang W., Liu T., Zhong Y., He G. (2024). A 5-Hydroxymethylcytosine-Based Non-Invasive Model for Early Detection of Colorectal Carcinomas and Advanced Adenomas: The METHOD-2 Study. Clin. Cancer Res. Off. J. Am. Assoc. Cancer Res..

[138] Fu Y.-L., Wu Y.-H., Cao D.-H., Jia Z.-F., Shen A., Jiang J., Cao X.-Y. (2022). Increased 5-Hydroxymethylcytosine Is a Favorable Prognostic Factor of Helicobacter Pylori-Negative Gastric Cancer Patients. World J. Gastrointest. Oncol..

[139] Kuang L., Zhang J., Li Y., Wang Q., Liu J., Zhang B. (2024). Association of Tet Methylcytosine Dioxygenase 2 and 5-Hydroxymethylcytosine in Endometrioid Adenocarcinoma and Its Clinical Significance. BMC Women’s Health.

[140] Shao J., Olsen R.J., Kasparian S., He C., Bernicker E.H., Li Z. (2024). Cell-Free DNA 5-Hydroxymethylcytosine Signatures for Lung Cancer Prognosis. Cells.

[141] Guo X.-J., Huang X.-Y., Yang X., Lu J.-C., Wei C.-Y., Gao C., Pei Y.-Z., Chen Y., Sun Q.-M., Cai J.-B. (2023). Loss of 5-Hydroxymethylcytosine Induces Chemotherapy Resistance in Hepatocellular Carcinoma via the 5-hmC/PCAF/AKT Axis. Cell Death Dis..

[142] Chen H.-Y., Zhang W.-L., Zhang L., Yang P., Li F., Yang Z.-R., Wang J., Pang M., Hong Y., Yan C. (2021). 5-Hydroxymethylcytosine Profiles of cfDNA Are Highly Predictive of R-CHOP Treatment Response in Diffuse Large B Cell Lymphoma Patients. Clin. Epigenet..

[143] Guler G.D., Ning Y., Coruh C., Mognol G.P., Phillips T., Nabiyouni M., Hazen K., Scott A., Volkmuth W., Levy S. (2024). Plasma Cell-Free DNA Hydroxymethylation Profiling Reveals Anti-PD-1 Treatment Response and Resistance Biology in Non-Small Cell Lung Cancer. J. Immunother. Cancer.

[144] Zhang Z., Pi X., Gao C., Zhang J., Xia L., Yan X., Hu X., Yan Z., Zhang S., Wei A. (2023). Integrated Fragmentomic Profile and 5-Hydroxymethylcytosine of Capture-Based Low-Pass Sequencing Data Enables Pan-Cancer Detection via cfDNA. Transl. Oncol..

[145] Shi Z.-D., Han X.-X., Song Z.-J., Dong Y., Pang K., Wang X.-L., Liu X.-Y., Lu H., Xu G.-Z., Hao L. (2023). Integrative Multi-Omics Analysis Depicts the Methylome and Hydroxymethylome in Recurrent Bladder Cancers and Identifies Biomarkers for Predicting PD-L1 Expression. Biomark. Res..

[146] Kilgour E., Rothwell D.G., Brady G., Dive C. (2020). Liquid Biopsy-Based Biomarkers of Treatment Response and Resistance. Cancer Cell.

[147] Zhang S., Zhang J., Hu X., Yin S., Yuan Y., Xia L., Cao F., Yan X., Yan Z., Mao Q. (2023). Noninvasive Detection of Brain Gliomas Using Plasma Cell-Free DNA 5-Hydroxymethylcytosine Sequencing. Int. J. Cancer.

[148] Storebjerg T.M., Strand S.H., Høyer S., Lynnerup A.-S., Borre M., Ørntoft T.F., Sørensen K.D. (2018). Dysregulation and Prognostic Potential of 5-Methylcytosine (5mC), 5-Hydroxymethylcytosine (5hmC), 5-Formylcytosine (5fC), and 5-Carboxylcytosine (5caC) Levels in Prostate Cancer. Clin. Epigenet..

[149] Azizgolshani N., Petersen C.L., Chen Y., Levy J.J., Salas L.A., Perreard L., Nguyen L.N., Christensen B.C. (2021). DNA 5-Hydroxymethylcytosine in Pediatric Central Nervous System Tumors May Impact Tumor Classification and Is a Positive Prognostic Marker. Clin. Epigenet..

[150] Shen Y., Wang L., Ou J., Wang B., Cen X. (2022). Loss of 5-Hydroxymethylcytosine as a Poor Prognostic Factor for Primary Testicular Diffuse Large B-Cell Lymphoma. Int. J. Med. Sci..

[151] Sharma A.E., Olivas A., Parilla M., Yassan L., Wang H., Zhang S.S., Weber C., Keutgen X.M., Hart J., Krausz T. (2022). Epigenetic Dysregulation of 5-Hydroxymethylcytosine in Well-Differentiated Pancreatic Neuroendocrine Tumors. Appl. Immunohistochem. Mol. Morphol. AIMM.

[152] Wu H.-X., Chen Y.-X., Wang Z.-X., Zhao Q., He M.-M., Wang Y.-N., Wang F., Xu R.-H. (2019). Alteration in TET1 as Potential Biomarker for Immune Checkpoint Blockade in Multiple Cancers. J. Immunother. Cancer.

[153] Peng D., He A., He S., Ge G., Wang S., Ci W., Li X., Xia D., Zhou L. (2022). Ascorbic Acid Induced TET2 Enzyme Activation Enhances Cancer Immunotherapy Efficacy in Renal Cell Carcinoma. Int. J. Biol. Sci..

[154] Liu N., Zhang J., Yan M., Chen L., Wu J., Tao Q., Yan B., Chen X., Peng C. (2023). Supplementation with α-Ketoglutarate Improved the Efficacy of Anti-PD1 Melanoma Treatment through Epigenetic Modulation of PD-L1. Cell Death Dis..

[155] Doroshow D.B., Sanmamed M.F., Hastings K., Politi K., Rimm D.L., Chen L., Melero I., Schalper K.A., Herbst R.S. (2019). Immunotherapy in Non-Small Cell Lung Cancer: Facts and Hopes. Clin. Cancer Res. Off. J. Am. Assoc. Cancer Res..

[156] Chakraborty S., Sharma G., Karmakar S., Banerjee S. (2024). Multi-OMICS Approaches in Cancer Biology: New Era in Cancer Therapy. Biochim. Biophys. Acta Mol. Basis Dis..

[157] Penny L., Main S.C., De Michino S.D., Bratman S.V. (2024). Chromatin- and Nucleosome-Associated Features in Liquid Biopsy: Implications for Cancer Biomarker Discovery. Biochem. Cell Biol..

[158] Hudecova I., Smith C.G., Hänsel-Hertsch R., Chilamakuri C.S., Morris J.A., Vijayaraghavan A., Heider K., Chandrananda D., Cooper W.N., Gale D. (2022). Characteristics, Origin, and Potential for Cancer Diagnostics of Ultrashort Plasma Cell-Free DNA. Genome Res..

[159] Chen L., Abou-Alfa G.K., Zheng B., Liu J.-F., Bai J., Du L.-T., Qian Y.-S., Fan R., Liu X.-L., Wu L. (2021). Genome-Scale Profiling of Circulating Cell-Free DNA Signatures for Early Detection of Hepatocellular Carcinoma in Cirrhotic Patients. Cell Res..

[160] Walker N.J., Rashid M., Yu S., Bignell H., Lumby C.K., Livi C.M., Howell K., Morley D.J., Morganella S., Barrell D. (2022). Hydroxymethylation Profile of Cell-Free DNA Is a Biomarker for Early Colorectal Cancer. Sci. Rep..

[161] Kamdar S.N., Ho L.T., Kron K.J., Isserlin R., van der Kwast T., Zlotta A.R., Fleshner N.E., Bader G., Bapat B. (2016). Dynamic Interplay between Locus-Specific DNA Methylation and Hydroxymethylation Regulates Distinct Biological Pathways in Prostate Carcinogenesis. Clin. Epigenet..

[162] Dang Y., Xu R., Pan J., Xiao X., Zhang S., Zhou W., Xu Y., Ji G. (2023). Dynamic Changes in DNA Methylation and Hydroxymethylation Revealed the Transformation of Advanced Adenoma into Colorectal Carcinoma. Clin. Transl. Med..

[163] Delhommeau F., Dupont S., Della Valle V., James C., Trannoy S., Massé A., Kosmider O., Le Couedic J.-P., Robert F., Alberdi A. (2009). Mutation in TET2 in Myeloid Cancers. N. Engl. J. Med..

[164] Abdel-Wahab O., Mullally A., Hedvat C., Garcia-Manero G., Patel J., Wadleigh M., Malinge S., Yao J., Kilpivaara O., Bhat R. (2009). Genetic Characterization of TET1, TET2, and TET3 Alterations in Myeloid Malignancies. Blood.

[165] Ko M., Huang Y., Jankowska A.M., Pape U.J., Tahiliani M., Bandukwala H.S., An J., Lamperti E.D., Koh K.P., Ganetzky R. (2010). Impaired Hydroxylation of 5-Methylcytosine in Myeloid Cancers with Mutant TET2. Nature.

[166] Lian C.G., Xu Y., Ceol C., Wu F., Larson A., Dresser K., Xu W., Tan L., Hu Y., Zhan Q. (2012). Loss of 5-Hydroxymethylcytosine Is an Epigenetic Hallmark of Melanoma. Cell.

[167] Boudra R., Woappi Y., Wang D., Xu S., Wells M., Schmults C.D., Lian C.G., Ramsey M.R. (2022). Regulation of 5-Hydroxymethylcytosine by TET2 Contributes to Squamous Cell Carcinoma Tumorigenesis. J. Investig. Dermatol..

[168] Alrehaili A.A., Gharib A.F., Alghamdi S.A., Alhazmi A., Al-Shehri S.S., Hagag H.M., Alsaeedi F.A., Alhuthali H.M., Raafat N., Etewa R.L. (2023). Evaluation of TET Family Gene Expression and 5-Hydroxymethylcytosine as Potential Epigenetic Markers in Non-Small Cell Lung Cancer. In Vivo.

[169] Cheng G., Wu J., Ji M., Hu W., Wu C., Jiang J. (2023). TET2 Inhibits the Proliferation and Metastasis of Lung Adenocarcinoma Cells via Activation of the cGAS-STING Signalling Pathway. BMC Cancer.

[170] Pavlovic V., Ciric M., Petkovic M., Golubovic M. (2023). Vitamin C and Epigenetics: A Short Physiological Overview. Open Med..

[171] Jiang X., Hu C., Ferchen K., Nie J., Cui X., Chen C.-H., Cheng L., Zuo Z., Seibel W., He C. (2017). Targeted Inhibition of STAT/TET1 Axis as a Therapeutic Strategy for Acute Myeloid Leukemia. Nat. Commun..

[172] Choudhury S.R., Cui Y., Lubecka K., Stefanska B., Irudayaraj J. (2016). CRISPR-dCas9 Mediated TET1 Targeting for Selective DNA Demethylation at BRCA1 Promoter. Oncotarget.

[173] Morita S., Noguchi H., Horii T., Nakabayashi K., Kimura M., Okamura K., Sakai A., Nakashima H., Hata K., Nakashima K. (2016). Targeted DNA Demethylation in Vivo Using dCas9-Peptide Repeat and scFv-TET1 Catalytic Domain Fusions. Nat. Biotechnol..

[174] Xu X., Tan X., Tampe B., Wilhelmi T., Hulshoff M.S., Saito S., Moser T., Kalluri R., Hasenfuss G., Zeisberg E.M. (2018). High-Fidelity CRISPR/Cas9- Based Gene-Specific Hydroxymethylation Rescues Gene Expression and Attenuates Renal Fibrosis. Nat. Commun..

[175] Nguyen T.V., Lister R. (2021). Genomic Targeting of TET Activity for Targeted Demethylation Using CRISPR/Cas9. Methods Mol. Biol..

[176] Bayliak M.M., Lushchak V.I. (2021). Pleiotropic Effects of α-Ketoglutarate as a Potential Anti-Ageing Agent. Ageing Res. Rev..

[177] Xu W., Yang H., Liu Y., Yang Y., Wang P., Kim S.-H., Ito S., Yang C., Wang P., Xiao M.-T. (2011). Oncometabolite 2-Hydroxyglutarate Is a Competitive Inhibitor of α-Ketoglutarate-Dependent Dioxygenases. Cancer Cell.

[178] Han S., Liu Y., Cai S.J., Qian M., Ding J., Larion M., Gilbert M.R., Yang C. (2020). IDH Mutation in Glioma: Molecular Mechanisms and Potential Therapeutic Targets. Br. J. Cancer.

[179] Jin S.-G., Jiang Y., Qiu R., Rauch T.A., Wang Y., Schackert G., Krex D., Lu Q., Pfeifer G.P. (2011). 5-Hydroxymethylcytosine Is Strongly Depleted in Human Cancers but Its Levels Do Not Correlate with IDH1 Mutations. Cancer Res..

[180] Minor E.A., Court B.L., Young J.I., Wang G. (2013). Ascorbate Induces Ten-Eleven Translocation (Tet) Methylcytosine Dioxygenase-Mediated Generation of 5-Hydroxymethylcytosine. J. Biol. Chem..

[181] Yin R., Mao S.-Q., Zhao B., Chong Z., Yang Y., Zhao C., Zhang D., Huang H., Gao J., Li Z. (2013). Ascorbic Acid Enhances Tet-Mediated 5-Methylcytosine Oxidation and Promotes DNA Demethylation in Mammals. J. Am. Chem. Soc..

[182] Agathocleous M., Meacham C.E., Burgess R.J., Piskounova E., Zhao Z., Crane G.M., Cowin B.L., Bruner E., Murphy M.M., Chen W. (2017). Ascorbate Regulates Haematopoietic Stem Cell Function and Leukaemogenesis. Nature.

[183] Gustafson C.B., Yang C., Dickson K.M., Shao H., Van Booven D., Harbour J.W., Liu Z.-J., Wang G. (2015). Epigenetic Reprogramming of Melanoma Cells by Vitamin C Treatment. Clin. Epigenet..

[184] Kim H., Park K.U. (2023). Clinical Circulating Tumor DNA Testing for Precision Oncology. Cancer Res. Treat. Off. J. Korean Cancer Assoc..

[185] Fiala C., Diamandis E.P. (2018). Utility of Circulating Tumor DNA in Cancer Diagnostics with Emphasis on Early Detection. BMC Med..

[186] Kustanovich A., Schwartz R., Peretz T., Grinshpun A. (2019). Life and Death of Circulating Cell-Free DNA. Cancer Biol. Ther..

[187] van der Pol Y., Mouliere F. (2019). Toward the Early Detection of Cancer by Decoding the Epigenetic and Environmental Fingerprints of Cell-Free DNA. Cancer Cell.

[188] Webster J., Dang H.X., Chauhan P.S., Feng W., Shiang A., Harris P.K., Pachynski R.K., Chaudhuri A.A., Maher C.A. (2023). PACT: A Pipeline for Analysis of Circulating Tumor DNA. Bioinformatics.

[189] Ul Haq S., Schmid S., Aparnathi M.K., Hueniken K., Zhan L.J., Sacdalan D., Li J.J.N., Meti N., Patel D., Cheng D. (2022). Cell-Free DNA Methylation-Defined Prognostic Subgroups in Small Cell Lung Cancer Identified by Leukocyte Methylation Subtraction. iScience.

